# Supplementary material for: How to attract and retain health workers in rural areas of a fragile state: Findings from a labour market survey in Guinea
Source: PLoS One. 2021 Dec 16;16(12):e0245569. doi: 10.1371/journal.pone.0245569 (PMC8675729; doi:10.1371/journal.pone.0245569)
Supplement: S1 File — (PDF) [file pone.0245569.s001.pdf]

This PDF generated by hlaufer, 1/29/2018 5:49:13 AM
Questionnaire created by andreaskutka, 11/20/2017 9:32:43 PM
Last modified by andreaskutka, 11/22/2017 4:53:57 PM

Not shared with anyone

Sections: 12, Sub-sections: 12,
Questions: 241.
Questions with enabling conditions: 65
Questions with validation conditions:45
Rosters: 0
Variables: 1

Survey Solutions

Professionnels de santé

A ADMIN
No sub-sections, No rosters, Questions: 13, Variables: 1.

B CONSENT
No sub-sections, No rosters, Questions: 6, Static texts: 1.

C IDENTIFICATION
No sub-sections, No rosters, Questions: 3.

D JOB CHOICES
No sub-sections, No rosters, Questions: 35, Static texts: 21.

E CURRENT JOB
Sub-sections: 5, No rosters, Questions: 36.

F PREFERENCES
No sub-sections, No rosters, Questions: 10.

G JOB HISTORY
No sub-sections, No rosters, Questions: 25, Static texts: 3.

H INCOME AND EXPENDITURE
Sub-sections: 5, No rosters, Questions: 46, Static texts: 1.

I ABSENTEEISM
No sub-sections, No rosters, Questions: 10.

J SATISFACTION AND CONSTRAINTS
Sub-sections: 2, No rosters, Questions: 28.

K CHARACTERISTICS OF THE RESPONDENT
No sub-sections, No rosters, Questions: 25.

L END OF INTERVIEW
No sub-sections, No rosters, Questions: 4.

LEGEND

A ADMIN

ID code of the health facility	TEXT<br>SCOPE: IDENTIFYING<br>..	hfid
ID code of the interviewer	TEXT<br>..	a1
ID code of the supervisor	TEXT<br>..	a2
Interview date	DATE: CURRENT TIME<br>..	a3
Name of the health facility	TEXT<br>..	a4
Type of health facility	SINGLE-SELECT<br>01 ○ National Hospital<br>02 ○ Regional Hospital<br>03 ○ Prefectural Hospital<br>04 ○ Communal Hospital<br>05 ○ Improved Health Center<br>06 ○ Health Center<br>07 ○ Health Post	a5
Location of the facility	SINGLE-SELECT<br>01 ○ Urban<br>02 ○ Rural	a6
Zone of the facility	SINGLE-SELECT<br>01 ○ Zone 1<br>02 ○ Zone 2<br>03 ○ Zone 3<br>04 ○ Zone 4<br>98 ○ I don’t know	a7
Region of the facility	SINGLE-SELECT<br>01 ○ Boké<br>02 ○ Conakry<br>03 ○ Faranah<br>04 ○ Kankan<br>05 ○ Kindia<br>06 ○ Labé<br>07 ○ Mamou<br>08 ○ Nzérékoré	a8
Prefectures	TEXT<br>..	a9
Sub-Prefectures	TEXT<br>..	a10
Nearest town (geographical)	TEXT<br>..	a11

GPS coordinates	GPS<br>N<br>W<br>A	a12
VARIABLE<br>Quest.IRnd()	DOUBLE	rnd

B CONSENT

STATIC TEXT

Hello. My name is ________________________ and I am from CERREGUI. We are working with the Ministry of Health, the World Bank and Oxford Policy Management on this health analytical project. We would like to know how you became a health worker, the challenges you face in your daily work, about your motivations and preferences. Your answers will help us better understand your situation as a health worker and we will present the findings to the Ministry of Health. This can help the policy makers design better policies for health workers in Guinea. The interview would take approximately 60 minutes. Participation in this research is voluntary. We will report the findings in a way that nobody will know what you tell me today. We will present the aggregate results without the details of what each patient is telling us. That means that you can be completely honest with us. Of course you can refuse to answer any questions and stop the interview at any time if you wish to. At this point, do you have any questions about the study? Do I have your agreement to proceed?

I understand that the information I will provide in the questionnaire includes identifiers, so that I can be contacted for clarification. I understand that the identifier information is not used in the analysis; each form will be anonymised and no personal information will be shared with anyone else, but the research team.<br>V1 self==1<br>M1 EXPLAIN TO RESPONDENT IN SIMPLE TERMS	SINGLE-SELECT<br>01 ○ Yes<br>02 ○ No	b1
I understand that my participation is voluntary and that I am free to withdraw at any time without giving any reason, without my medical care or legal rights being affected.<br>V1 self==1<br>M1 EXPLAIN TO RESPONDENT IN SIMPLE TERMS	SINGLE-SELECT<br>01 ○ Yes<br>02 ○ No	b2
I understand that the information from my interview will be pooled with other participants' responses, anonymised and may be published.<br>V1 self==1<br>M1 EXPLAIN TO RESPONDENT IN SIMPLE TERMS	SINGLE-SELECT<br>01 ○ Yes<br>02 ○ No	b3
Do you want to participate in our survey?<br>E b1==1 && b2==1 && b3==1	SINGLE-SELECT<br>01 ○ Yes<br>02 ○ No	b4
If you are not willing to participate in this survey would you please tell us the main reason for not participating in this survey?<br>E b4==2	SINGLE-SELECT<br>01 ○ Do not have time<br>02 ○ Do not have interest<br>03 ○ Not approved by my superiors<br>04 ○ Do not want to share personal information<br>05 ○ Fear/anxiety	b5

97 Others, specify
98 Don’t know

Specify other — TEXT — b5_oth

E b5==97

....

C IDENTIFICATION

Surname and name of the respondent — TEXT — c1

....

What is your official designation? — SINGLE-SELECT — c2

01 Specialist doctor
02 Doctor
03 Nurse
04 Midwife
05 ATS

Mobile phone number of the respondent — TEXT — c3

I Enter 000000000 if none

....

D JOB CHOICES

STATIC TEXT

A LIRE: Nous allons maintenant procéder a un exercice au cours duquel vous vous verrez proposer deux postes hypothétiques. Chaque poste a des caractéristiques différentes et vous devrez choisir le poste qui vous parait le meilleur des deux. Puis une sous question vous demandera si, en les conditions actuelles, vous accepteriez en effet le poste en question. D’abord, merci de prendre quelques minutes pour vous familiariser avec les caractéristiques des postes

STATIC TEXT

A MONTRER:
(1) la feuille avec les differentes characteristiques en rapport avec le type de travailleur: %c2%
(2) L'exemple pour le travailleur %c2%

STATIC TEXT

E (c2==1 || c2 == 2) && rnd<0.5

Procédure alternative: Veuillez passer la tablette à l'enqueté(e) et faire exemple avec lui/elle

Médecins: EXEMPLE

Caractéristiques	Poste A	Poste B
Salaire	6.5 millions	2 millions
Localisation	Conakry	capitale préfectorale
Equipement	Bon	Mediocre
Formation	spécialisation	aucun
Temps d'affectation	indéterminée	indéterminée
Logement	logement	aucun
Transport	moto	aucun

Exemple: faites le choix — SINGLE-SELECT — choice_ex

E (c2==1 || c2 == 2) && rnd<0.5

01 Job A
02 Job B

STATIC TEXT

E rnd>=0.5 || c2==3 || c2 == 4 || c2 == 5

Mettre l'heure de depart	DATE: CURRENT TIME DCE_start_time …………………………………………

STATIC TEXT

E (c2==1 || c2 == 2) && rnd<0.5

Choix Numéro 1

Caractéristiques	Poste A	Poste B
Salaire	6.5 millions	6.5 millions
Localisation	capitale préfectorale	Conakry
Equipement	Médiocre	bon
Formation	spécialisation	ateliers
Temps d'affectation	indéterminée	indéterminée
Logement	aucun	aucun
Transport	moto	aucun

Choice Set Number 1	SINGLE-SELECT choice1<br>01 ○ Job A<br>02 ○ Job B
Si vous pouviez avoir ce poste, l'accepteriez-vous?	SINGLE-SELECT choice1_would<br>01 ○ oui<br>02 ○ non

STATIC TEXT

E (c2==1 || c2 == 2) && rnd<0.5

Choix Numéro 2

Caractéristiques	Poste A	Poste B
Salaire	3.5 millions	2 millions
Localisation	capitale préfectorale	capitale sous-préfectorale (CS)
Equipement	moyen	médiocre
Formation	ateliers	ateliers
Temps d'affectation	indéterminée	5 ans
Logement	aucun	logement
Transport	moto	aucun

Choice Set Number 2	SINGLE-SELECT choice2<br>01 ○ Job A<br>02 ○ Job B
Si vous pouviez avoir ce poste, l'accepteriez-vous?	SINGLE-SELECT choice2_would<br>01 ○ oui<br>02 ○ non

STATIC TEXT

E (c2==1 || c2 == 2) && rnd<0.5

Choix Numéro 3

Caractéristiques	Poste A	Poste B
Salaire	2 millions	3.5 millions
Localisation	Conakry	capitale préfectorale
Equipement	moyen	moyen
Formation	spécialisation	ateliers
Temps d'affectation	indéterminée	indéterminée
Logement	aucun	logement
Transport	aucun	moto

Choice Set Number 3

SINGLE-SELECT choice3

01 Job A
02 Job B

Si vous pouviez avoir ce poste, l'accepteriez-vous?

SINGLE-SELECT choice3_would

01 oui
02 non

STATIC TEXT

E (c2==1 || c2 == 2) && rnd<0.5

Choix Numéro 4

Caractéristiques	Poste A	Poste B
Salaire	2 millions	6.5 millions
Localisation	capitale préfectorale	capitale sous-préfectorale (CS)
Equipement	bon	moyen
Formation	aucun	aucun
Temps d'affectation	indéterminée	5 ans
Logement	logement	aucun
Transport	aucun	moto

Choice Set Number 4

SINGLE-SELECT choice4

01 Job A
02 Job B

Si vous pouviez avoir ce poste, l'accepteriez-vous?

SINGLE-SELECT choice4_would

01 oui
02 non

STATIC TEXT

E (c2==1 || c2 == 2) && rnd<0.5

Choix Numéro 5

Caractéristiques	Poste A	Poste B
Salaire	3.5 millions	2 millions
Localisation	Conakry	capitale sous-préfectorale (CS)
Equipement	médiocre	médiocre
Formation	aucun	ateliers
Temps d'affectation	indéterminée	indéterminée
Logement	aucun	aucun
Transport	aucun	moto

Choice Set Number 5

SINGLE-SELECT choice5

01 Job A
02 Job B

Si vous pouviez avoir ce poste, l'accepteriez-vous?

SINGLE-SELECT choice5_would

01 oui
02 non

STATIC TEXT

E (c2==1 || c2 == 2) && rnd<0.5

Choix Numéro 6

Caractéristiques	Poste A	Poste B
Salaire	2 millions	2 millions
Localisation	Conakry	capitale préfectorale
Equipement	moyen	bon
Formation	spécialisation	aucun
Temps d'affectation	indéterminée	indéterminée
Logement	aucun	aucun
Transport	aucun	moto

Choice Set Number 6	SINGLE-SELECT choice6<br>01 ○ Job A<br>02 ○ Job B
Si vous pouviez avoir ce poste, l'accepteriez-vous?	SINGLE-SELECT choice6_would<br>01 ○ oui<br>02 ○ non

STATIC TEXT

E (c2==1 || c2 == 2) && rnd<0.5

Choix Numéro 7

Caractéristiques	Poste A	Poste B
Salaire	3.5 millions	3.5 millions
Localisation	capitale préfectorale	capitale sous-préfectorale (CS)
Equipement	moyen	bon
Formation	ateliers	spécialisation
Temps d'affectation	indéterminée	indéterminée
Logement	aucun	logement
Transport	moto	aucun

Choice Set Number 7	SINGLE-SELECT choice7<br>01 ○ Job A<br>02 ○ Job B
Si vous pouviez avoir ce poste, l'accepteriez-vous?	SINGLE-SELECT choice7_would<br>01 ○ oui<br>02 ○ non

STATIC TEXT

E (c2==1 || c2 == 2) && rnd<0.5

Choix Numéro 8

Caractéristiques	Poste A	Poste B
Salaire	3.5 millions	2 millions
Localisation	capitale sous-préfectorale (CS)	capitale préfectorale
Equipement	bon	bon
Formation	spécialisation	aucun
Temps d'affectation	indéterminée	5 ans
Logement	aucun	logement
Transport	aucun	moto

Choice Set Number 8	SINGLE-SELECT choice8<br>01 ○ Job A<br>02 ○ Job B
Si vous pouviez avoir ce poste, l'accepteriez-vous?	SINGLE-SELECT choice8_would<br>01 ○ oui<br>02 ○ non

STATIC TEXT

E (c2==1 || c2 == 2) && rnd<0.5

Choix Numéro 9

Caractéristiques	Poste A	Poste B
Salaire	6.5 millions	2 millions
Localisation	capitale sous-préfectorale (CS)	Conakry
Equipement	moyen	moyen
Formation	aucun	spécialisation
Temps d'affectation	5 ans	indéterminée
Logement	aucun	aucun
Transport	aucun	aucun

Choice Set Number 9	SINGLE-SELECT choice9<br>01 Job A<br>02 Job B
Si vous pouviez avoir ce poste, l'accepteriez-vous?	SINGLE-SELECT choice9_would<br>01 oui<br>02 non

STATIC TEXT

E (c2==1 || c2 == 2) && rnd<0.5

Choix Numéro 10

Caractéristiques	Poste A	Poste B
Salaire	6.5 millions	3.5 millions
Localisation	Conakry	capitale préfectorale
Equipement	bon	moyen
Formation	ateliers	ateliers
Temps d'affectation	indéterminée	5 ans
Logement	aucun	logement
Transport	aucun	aucun

Choice Set Number 10	SINGLE-SELECT choice10<br>01 Job A<br>02 Job B
Si vous pouviez avoir ce poste, l'accepteriez-vous?	SINGLE-SELECT choice10_would<br>01 oui<br>02 non

STATIC TEXT

E (c2==1 || c2 == 2) && rnd<0.5

Choix Numéro 11

Caractéristiques	Poste A	Poste B
Salaire	3.5 millions	3.5 millions
Localisation	capitale sous-préfectorale (CS)	Conakry
Equipement	bon	médiocre
Formation	spécialisation	aucun
Temps d'affectation	indéterminée	indéterminée
Logement	aucun	aucun
Transport	aucun	aucun

Choice Set Number 11	SINGLE-SELECT choice11<br>01 Job A<br>02 Job B
Si vous pouviez avoir ce poste, l'accepteriez-vous?	SINGLE-SELECT choice11_would<br>01 oui

02 non

STATIC TEXT

E (c2==1 || c2 == 2) && rnd<0.5

Choix Numéro 12

Caractéristiques	Poste A	Poste B
Salaire	3.5 millions	6.5 millions
Localisation	Conakry	capitale sous-préfectorale (CS)
Equipement	médiocre	moyen
Formation	aucun	aucun
Temps d'affectation	indéterminée	5 ans
Logement	aucun	logement
Transport	aucun	aucun

Choice Set Number 12

SINGLE-SELECT choice12

01 Job A
02 Job B

Si vous pouviez avoir ce poste, l'accepteriez-vous?

SINGLE-SELECT choice12_would

01 oui
02 non

STATIC TEXT

E (c2==1 || c2 == 2) && rnd<0.5

Choix Numéro 13

Caractéristiques	Poste A	Poste B
Salaire	2 millions	2 millions
Localisation	capitale sous-préfectorale (CS)	capitale préfectorale
Equipement	médiocre	bon
Formation	ateliers	aucun
Temps d'affectation	indéterminée	5 ans
Logement	logement	aucun
Transport	moto	aucun

Choice Set Number 13

SINGLE-SELECT choice13

01 Job A
02 Job B

Si vous pouviez avoir ce poste, l'accepteriez-vous?

SINGLE-SELECT choice13_would

01 oui
02 non

STATIC TEXT

E (c2==1 || c2 == 2) && rnd<0.5

Choix Numéro 14

Caractéristiques	Poste A	Poste B
Salaire	6.5 millions	3.5 millions
Localisation	capitale sous-préfectorale (CS)	capitale préfectorale
Equipement	moyen	moyen
Formation	aucun	ateliers
Temps d'affectation	indéterminée	5 ans
Logement	logement	aucun
Transport	moto	aucun

Choice Set Number 14

SINGLE-SELECT choice14

01 Job A
02 Job B

Si vous pouviez avoir ce poste, l'accepteriez-vous?	SINGLE-SELECT choice14_would<br>01 ○ oui<br>02 ○ non

STATIC TEXT

E (c2==1 || c2 == 2) && rnd<0.5

Choix Numéro 15

Caractéristiques	Poste A	Poste B
Salaire	6.5 millions	6.5 millions
Localisation	capitale préfectorale	capitale sous-préfectorale (CS)
Equipement	médiocre	moyen
Formation	spécialisation	aucun
Temps d'affectation	5 ans	indéterminée
Logement	aucun	logement
Transport	moto	aucun

Choice Set Number 15	SINGLE-SELECT choice15<br>01 ○ Job A<br>02 ○ Job B
Si vous pouviez avoir ce poste, l'accepteriez-vous?	SINGLE-SELECT choice15_would<br>01 ○ oui<br>02 ○ non

STATIC TEXT

E (c2==1 || c2 == 2) && rnd<0.5

Choix Numéro 16

Caractéristiques	Poste A	Poste B
Salaire	6.5 millions	3.5 millions
Localisation	capitale préfectorale	capitale sous-préfectorale (CS)
Equipement	médiocre	bon
Formation	spécialisation	spécialisation
Temps d'affectation	indéterminée	5 ans
Logement	logement	aucun
Transport	aucun	moto

Choice Set Number 16	SINGLE-SELECT choice16<br>01 ○ Job A<br>02 ○ Job B
Si vous pouviez avoir ce poste, l'accepteriez-vous?	SINGLE-SELECT choice16_would<br>01 ○ oui<br>02 ○ non

STATIC TEXT

E (c2==1 || c2 == 2) && rnd<0.5

Merci ! Veuillez rendre la tablette a l'enquêteur

Mettre l'heure de fin	DATE: CURRENT TIME DCE_end_time<br>..

E CURRENT JOB

E CURRENT JOB

How many years have you worked in your current position in this health facility?<br>I Write in completed years. If less than 1 year, write 0.<br>V1 `self.InRange(0,70)`<br>M1 Not in Range, It should be in between 0 to 70. Please confirm & Reente r.	NUMERIC: INTEGER — e4<br>________________
Did you want to be posted in this facility?	SINGLE-SELECT — e5<br>01 ○ Yes<br>02 ○ No
How many years have you worked in this facility?<br>I Write in completed years. If less than 1 year, write 0.<br>V1 `self.InRange(0,70)`<br>M1 Not in Range, It should be in between 0 to 70. Please confirm & Reente r.<br>V2 `self>=e4`<br>M2 Cannot be smaller than years in current position.	NUMERIC: INTEGER — e1<br>________________
In how many different positions have you worked in this facility?<br>I Write number of positions. If only one position, write 1<br>V1 `self.InRange(1,20)`	NUMERIC: INTEGER — e1_1<br>________________
Are you a permanent or contractual employee?<br>I Do not prompt.	SINGLE-SELECT — e2<br>01 ○ Public, permanent employee<br>02 ○ Contractual staff (municipality)<br>03 ○ Contractual staff (health facility)<br>04 ○ Contractual staff (NGO/programme-based)
What would you say is your current function?	SINGLE-SELECT — e3<br>01 ○ Management<br>02 ○ Medical/technical role<br>03 ○ Support role<br>04 ○ Many roles<br>97 ○ Others, specify
Specify other<br>E `e3==97`	TEXT — e3_oth<br>................................
Were you trained in this region?	SINGLE-SELECT — e6<br>01 ○ Yes<br>02 ○ No
Where were you trained?<br>E `e6==2`	SINGLE-SELECT — e7<br>01 ○ Boké<br>02 ○ Conakry<br>03 ○ Faranah<br>04 ○ Kankan<br>05 ○ Kindia<br>06 ○ Labé<br>07 ○ Mamou<br>08 ○ Nzérékoré
Did you practise in rural areas during the training?	SINGLE-SELECT — g6<br>01 ○ Yes<br>02 ○ No

Are you sometimes performing tasks which you were not prepared for in health professional training?<br><br>I Do not prompt.	SINGLE-SELECT e8<br>01 ○ Yes, daily<br>02 ○ Often<br>03 ○ Sometimes<br>04 ○ Never<br>99 ○ Prefer not say
Which tasks that you are performing now were you not trained for?<br><br>I Do not prompt. Select multiple.<br>E `e8==1 || e8==2`	MULTI-SELECT e9<br>01 ☐ Outpatient consultation<br>02 ☐ Accident and emergency<br>03 ☐ Minor surgery<br>04 ☐ Immunization<br>05 ☐ Maternal health services/ advice<br>06 ☐ Family planning services/ advice<br>07 ☐ Pharmacy<br>97 ☐ Others, specify
Specify other<br><br>E `e9.Contains(97)`	TEXT e9 oth<br>..
Overall, do you feel well prepared and trained for your current position?<br><br>I Do not prompt.	SINGLE-SELECT e10<br>01 ○ Yes<br>02 ○ Sometimes, I feel overwhelmed<br>03 ○ Not at all

E CURRENT JOB

ACTIVITIES AND RESPONSIBILITIES

Do you consult patients daily?	SINGLE-SELECT e11<br>01 ○ Yes, daily<br>02 ○ Often<br>03 ○ Sometimes<br>04 ○ Never
Why not?<br><br>E `e11==4`	SINGLE-SELECT e12<br>01 ○ Only few patients visit this facility<br>02 ○ I am doing other tasks<br>97 ○ Others, specify
Specify other<br><br>E `e12==97`	TEXT e12oth<br>..
On average, how many patients do you consult in a day, when you are at this facility?<br><br>E `e11==1 || e11==2 || e11==3`<br>V1 `self.InRange(1,80)`<br>M1 Out of range	NUMERIC: INTEGER e13<br>_ _ _ _ _ _ _ _ _ _ _ _ _ _ _ _ _ _ _ _
How do you feel about the number of patients you are seeing in a day?<br><br>I Prompt.<br>E `e11==1 || e11==2 || e11==3`	SINGLE-SELECT e14<br>01 ○ More than I can take care of<br>02 ○ Just enough patients<br>03 ○ Could handle a few more patients<br>04 ○ Could handle a lot more patients

Question	Response
Can you meet the needs of the patients you are consulting?<br><br>E e11==1 || e11==2	SINGLE-SELECT e15<br>01 ○ Yes<br>02 ○ No
Why not?<br><br>I Do not prompt.<br>E (e11==1 || e11==2 || e11==3) && e15==2	SINGLE-SELECT e16<br>01 ○ The facility only offers limited services<br>02 ○ Lack of equipment<br>03 ○ Broken equipment<br>04 ○ Insufficient training/skills<br>05 ○ Lack of personnel<br>97 ○ Others, specify
Specify other<br><br>E (e11==1 || e11==2 || e11==3) && e16==97	TEXT e16oth<br>..

E CURRENT JOB

WORKLOAD

Question	Response
In a typical week, how many days are you working at this facility?<br><br>I Write number of days. Write 0 if none.<br>V1 self.InRange(0,7)<br>M1 Out of Range	NUMERIC: INTEGER e17<br>_ _ _ _ _ _ _ _ _ _ _ _ _ _ _ _ _ _ _ _
Do you feel that your colleagues (same cadre) work roughly the same number of days as you do in a typical week in this facility?	SINGLE-SELECT e18<br>01 ○ They work more<br>02 ○ They work the same<br>03 ○ They work less<br>04 ○ Not applicable
In a typical day, how many hours do you work in this facility?<br><br>I Write the number of hours per day.<br>V1 self.InRange(0,24)<br>M1 Out of Range	NUMERIC: INTEGER e19<br>_ _ _ _ _ _ _ _ _ _ _ _ _ _ _ _ _ _ _ _
Do you feel that your colleagues (same cadre) work roughly the same number of hours in a typical day in this facility?	SINGLE-SELECT e20<br>01 ○ They work more<br>02 ○ They work the same<br>03 ○ They work less<br>99 ○ Not applicable
Do you feel as if you have sufficient time to complete all the tasks you have to complete?	SINGLE-SELECT e21<br>01 ○ Yes<br>02 ○ Sometimes I have trouble completing all<br>03 ○ No, I hardly manage to complete all tasks

E CURRENT JOB

MANAGEMENT AND SUPERVISION

Question	Response
Do you feel as if there is a strict hierarchy in your workspace?<br><br>I Explain 'hierarchy' as: little flexibility, staff in non-management positio ns has little decision-making power, top down communication from su perior, limited opportunities for feedback to superior	SINGLE-SELECT e22<br>01 ○ Yes<br>02 ○ No

Is your supervisor/superior generally present at the health facility you currently work at?	SINGLE-SELECT e23<br>01 ○ Yes<br>02 ○ No<br>97 ○ I don't know
What do you think would your supervisor/superior do if you did not come to work regularly or took leave without permission for an extended period?<br><br>I Select two.	MULTI-SELECT e24<br>01 ☐ Try to find out what is wrong<br>02 ☐ Report my behaviour<br>03 ☐ Motivate me to improve<br>04 ☐ Give a warning/describe the consequences<br>05 ☐ Withhold my salary<br>06 ☐ Sanctions, public accusation, suspension<br>07 ☐ I will lose my job<br>08 ☐ Nothing<br>97 ☐ Others, specify
Specify other<br><br>E `e24.Contains(97)`	TEXT e24oth<br>..
Do you feel as if your needs and suggestions are being considered by your supervisor/superior?	SINGLE-SELECT e25<br>01 ○ Yes<br>03 ○ Sometimes<br>02 ○ No<br>97 ○ I don't know

E CURRENT JOB

REFERRALS AND QUALITY OF SERVICES PROVIDED

Where do you refer patients to, if needed?	SINGLE-SELECT e26<br>01 ○ National Hospital<br>02 ○ Regional Hospital<br>03 ○ Prefectural Hospital<br>04 ○ Communal Hospital<br>05 ○ Improved Health Center<br>06 ○ Health Center<br>08 ○ Private Clinique
Do you feel that the health facility you are working at meets the demands of the patients?	SINGLE-SELECT e27<br>01 ○ Yes<br>02 ○ Sometimes<br>03 ○ No
If you could improve one of the following aspects in your health facility, which do you feel is the most important?<br><br>I Prompt.	SINGLE-SELECT e28<br>01 ○ Skills of staff<br>02 ○ Equipment<br>03 ○ Availability of medicine and materials<br>04 ○ Cleanliness and hygiene<br>05 ○ More staff<br>06 ○ Infrastructure<br>97 ○ Others, specify
Specify other<br><br>E `e28==97`	TEXT e28oth<br>..

F PREFERENCES

Question	Response
What was the main reason for you to become a health worker?<br>I Do not prompt.	SINGLE-SELECT f1<br>01 ○ To have a job<br>02 ○ To keep a job for a long time<br>03 ○ To have a job that pays well<br>04 ○ To access other income opportunities<br>05 ○ To have good social status<br>06 ○ To have a light workload<br>07 ○ To help others<br>08 ○ To continue family tradition<br>09 ○ To have great responsibilities<br>97 ○ Other, specify
Specify other<br>E f1==97	TEXT f1oth<br>..
Would you like a transfer from your current position in the near future? (In the next 1-2 years)	SINGLE-SELECT f2<br>01 ○ Yes<br>02 ○ No
In the longer term (3-5 years), do you plan to change profession?	SINGLE-SELECT f4<br>01 ○ Yes<br>02 ○ No
What is the reason for this?<br>E f4==1	SINGLE-SELECT f5<br>01 ○ Better income/salary<br>02 ○ Better career options<br>03 ○ I do not like my work<br>04 ○ Less stress<br>05 ○ Less responsibilities<br>97 ○ Other, specify
Specify other<br>E f5==97	TEXT f5 oth<br>..
In the longer term, would you prefer working in urban or rural areas?	SINGLE-SELECT f3<br>01 ○ Urban<br>02 ○ Rural
In the longer term, which sector would you prefer to work in?<br>E f4==2	SINGLE-SELECT f6<br>01 ○ Public sector/ government<br>02 ○ Private sector<br>03 ○ NGO sector<br>04 ○ Faith based sector
In the longer term, in which level of facility you would you prefer to work?<br>E f4==2	SINGLE-SELECT f7<br>01 ○ National Hospital<br>02 ○ Regional Hospital<br>03 ○ Prefectural Hospital<br>04 ○ Communal Hospital<br>05 ○ Improved Health Center<br>06 ○ Health Center<br>07 ○ Health Post
In the longer term, do you plan to migrate abroad?	SINGLE-SELECT f8<br>01 ○ Yes<br>02 ○ No

G JOB HISTORY

Question	Response	ID
How many years have you worked in your profession in total?<br>I Write in completed years. If less than 1 year, write 0.<br>V1 `self.InRange(0,70)`<br>M1 Out of range.	NUMERIC: INTEGER<br>_ _ _ _ _ _ _ _ _ _	g1
Have you worked as a health professional outside of Guinea?	SINGLE-SELECT<br>01 ○ Yes<br>02 ○ No	g2
For how many years?<br>I Write in completed years. If less than 1 year, write 0.<br>E `g2==1`<br>V1 `self.InRange(0,70)`<br>M1 Out of Range	NUMERIC: INTEGER<br>_ _ _ _ _ _ _ _ _ _	g3
Where?<br>I If more than one country, write country that respondent has worked in the longest time<br>E `g2==1`	TEXT<br>....................................	g4
How many years have you worked in your profession in Guinea?<br>I Write in completed years. If less than 1 year, write 0.<br>E `g2==1`<br>V1 `self.InRange(0,70)`<br>M1 Out of Range	NUMERIC: INTEGER<br>_ _ _ _ _ _ _ _ _ _	g5

STATIC TEXT

Training history

Question	Response	ID
Are you currently working close to the area, where you received your training/education as a health professional?	SINGLE-SELECT<br>01 ○ Yes<br>02 ○ No	g7
Are you currently working close to the area, where you grew up?	SINGLE-SELECT<br>01 ○ Yes<br>02 ○ No	g8

STATIC TEXT

Enumerator to give examples of urban and rural areas. Ensure respondent understands categories

Question	Response	ID
Have you worked in health facilities in urban areas?	SINGLE-SELECT<br>01 ○ Yes<br>02 ○ No	g9
For how many years?<br>I Write in completed years. If less than 1 year, write 0.<br>E `g9==1`<br>V1 `self.InRange(0,70)`<br>M1 Out of Range	NUMERIC: INTEGER<br>_ _ _ _ _ _ _ _ _ _	g10
Have you worked in health facilities in rural areas?	SINGLE-SELECT<br>01 ○ Yes<br>02 ○ No	g11
For how many years?<br>I Write in completed years. If less than 1 year, write 0.	NUMERIC: INTEGER<br>_ _ _ _ _ _ _ _ _ _	g12

E g11==1<br>V1 self.InRange(0,70)<br>M1 Out of Range	

STATIC TEXT

Enumerator to give local examples of national hospital, regional, prefectural and communal hospitals, health centres and health posts, the respondent can relate to. Ensure respondent understands categories.

Have you worked in one of the national hospitals?	SINGLE-SELECT g13<br>01 ○ Yes<br>02 ○ No
For how many years?<br>I Write in completed years. If less than 1 year, write 0.<br>E g13==1<br>V1 self.InRange(0,70)<br>M1 Out of Range	NUMERIC: INTEGER g14<br>_______________
Have you worked in regional hospitals?	SINGLE-SELECT g15<br>01 ○ Yes<br>02 ○ No
For how many years?<br>I Write in completed years. If less than 1 year, write 0.<br>E g15==1<br>V1 self.InRange(0,70)<br>M1 Out of Range	NUMERIC: INTEGER g16<br>_______________
Have you worked in prefectural hospitals or communal hospitals?	SINGLE-SELECT g17<br>01 ○ Yes<br>02 ○ No
For how many years?<br>I Write in completed years. If less than 1 year, write 0.<br>E g17==1<br>V1 self.InRange(0,70)<br>M1 Out of Range	NUMERIC: INTEGER g18<br>_______________
Have you worked in health centres?	SINGLE-SELECT g19<br>01 ○ Yes<br>02 ○ No
For how many years?<br>I Write in completed years. If less than 1 year, write 0.<br>E g19==1<br>V1 self.InRange(0,70)<br>M1 Out of Range	NUMERIC: INTEGER g20<br>_______________
Have you worked in a health post?	SINGLE-SELECT g21<br>01 ○ Yes<br>02 ○ No
For how many years?<br>I Write in completed years. If less than 1 year, write 0.<br>E g21==1<br>V1 self.InRange(0,70)<br>M1 Out of Range	NUMERIC: INTEGER g22<br>_______________
How many times have you changed health facility since you started to work in your	NUMERIC: INTEGER g23<br>_______________

profession?

I Write number of transfers. If no transfers, write 0

Question	Response	Variable
How many times have you changed position since you started to work in your profession?<br>I Write number of transfers. If no transfers, write 0	NUMERIC: INTEGER<br>_ _ _ _ _ _ _ _ _ _ _ _ _ _ _ _ _ _ _ _	g24
Have you worked in private health facilities?	SINGLE-SELECT<br>01 ◯ Yes<br>02 ◯ No	g25
For how many years?<br>I Write in completed years. If less than 1 year, write 0.<br>E `g25==1`<br>V1 `self.InRange(0,70)`<br>M1 Out of Range	NUMERIC: INTEGER<br>_ _ _ _ _ _ _ _ _ _ _ _ _ _ _ _ _ _ _ _	g26

H INCOME AND EXPENDITURE

H INCOME AND EXPENDITURE

INCOME

Question	Response	Variable
What is your gross salary per month?<br>I Record from payslip. Exclude all additional payments. Enter value in th ousand GNF 99998 I don't know<br>V1 `self.InRange(0,20000) || self==99998`<br>M1 Out of Range	NUMERIC: INTEGER<br>_ _ _ _ _ _ _ _ _ _ _ _ _ _ _ _ _ _ _ _	h1
What is your net (take home) salary per month?<br>I Record from pay slip. Exclude all additional payments. Enter value in th ousand GNF 99998 I don't know<br>V1 `self.InRange(0,20000) || self==99998`<br>M1 Out of Range	NUMERIC: INTEGER<br>_ _ _ _ _ _ _ _ _ _ _ _ _ _ _ _ _ _ _ _	h2

STATIC TEXT

In a normal month, how much do you receive of the following offical, additional payments?

INSTRUCTION
Enter value in thousand GNF. Record from payslip
Enter '0' if none

Question	Response	Variable
Transport allowance<br>V1 `self.InRange(0,2000)`<br>M1 Out of Range	NUMERIC: INTEGER<br>_ _ _ _ _ _ _ _ _ _ _ _ _ _ _ _ _ _ _ _	h7
Housing allowance<br>V1 `self.InRange(0,2000)`<br>M1 Out of Range	NUMERIC: INTEGER<br>_ _ _ _ _ _ _ _ _ _ _ _ _ _ _ _ _ _ _ _	h6
Other allowances (e.g. uniform allowance, medical allowance, etc.)<br>V1 `self.InRange(0,2000)`<br>M1 Out of Range	NUMERIC: INTEGER<br>_ _ _ _ _ _ _ _ _ _ _ _ _ _ _ _ _ _ _ _	h8

	Risk premium payments	NUMERIC: INTEGER	h10
V1	self.InRange(0,2000)		
M1	Out of Range		
	Per diem payments	NUMERIC: INTEGER	h12
V1	self.InRange(0,2000)		
M1	Out of Range		
	Performance based payments	NUMERIC: INTEGER	h14
V1	self.InRange(0,2000)		
M1	Out of range		
	Other, official payments (e.g rural hardship compensation, etc.)	NUMERIC: INTEGER	h15
V1	self.InRange(0,2000)		
M1	Out of range		
	Specify other payment	TEXT	h16
E	h15>0		
	During the last 12 months, how many times did you receive your salary late?	NUMERIC: INTEGER	h4
I	Write 0 if salary was always received on time		
V1	self.InRange(0,12)		
M1	Out of Range		
	During the last 12 months, how often did you NOT receive your salary AT ALL?	NUMERIC: INTEGER	h5
I	Write 0 if salary was ALWAYS received		
V1	self.InRange(0,12)		
M1	Out of Range		

H INCOME AND EXPENDITURE

USER FEES AND PRIVATE PRACTISE INCOME

	Is it a common practice to be paid by the patients, after delivering a service, when working in a public health facility?	SINGLE-SELECT<br>01 ○ Yes<br>02 ○ No	h17
	Are prices listed somewhere for the patient to see? (e.g. a price list for services on a wall or a sheet of paper)	SINGLE-SELECT<br>01 ○ Yes<br>03 ○ Some<br>02 ○ No	h17a
E	h17==1		
	Are patients informed in advance (before receiving the services) about the payments?	SINGLE-SELECT<br>01 ○ Yes<br>02 ○ No	h17b
E	h17==1		
	Is it a common practice to be paid by the patients, in order to deliver better or faster services, when working in a public health facility?	SINGLE-SELECT<br>01 ○ Yes<br>03 ○ Sometimes<br>02 ○ No<br>98 ○ Prefer not to say	h17c
	In a typical week, how much income do you think a health worker can receive from patient	NUMERIC: INTEGER	h18

payments received in a public health facility?

I Enter value in thousand GNF
E `h17==1`
V1 `self.InRange(0,5000)`
M1 Out of Range

How do you feel about patient payments in public health facilities?

I Do not prompt.
E `h17==1`

SINGLE-SELECT h19

01 ○ Uncomfortable
02 ○ Accepted
03 ○ Comfortable
97 ○ Others, specify

Specify other

E `h17==1 && h19==97`

TEXT h19oth

Is it a common practice to receive gifts from patients in public health facilities?

SINGLE-SELECT h20

01 ○ Yes
02 ○ No

Which kind of gifts do patients bring for health professionals working in public health facilities?

I DO NOT PROMPT, SELECT ALL THAT APPLIES
E `h20==1`

MULTI-SELECT h21

01 ☐ Vegetables/Staples/Fruit/Meat/Dairy
02 ☐ Fabric
03 ☐ Airtime vouchers
04 ☐ Cosmetics/Soap
97 ☐ Others, specify

Specify other

E `h20==1 && h21.Contains(97)`

TEXT h21oth

How do you feel about gift giving in public health facilities?

I Do not prompt.
E `h20==1`

SINGLE-SELECT h23

01 ○ Uncomfortable
02 ○ Accepted
03 ○ Comfortable
97 ○ Others, specify

Specify other

E `h23==97`

TEXT h23oth

Is it a common practice to privately consult patients outside of work hours?

SINGLE-SELECT h24

01 ○ Yes
02 ○ No

In a typical week, how much income do you think a health worker can receive from having a private practice outside of work hours?

I Enter value in GNF
E `h24==1`
V1 `self.InRange(0,50000)`
M1 Out of Range

NUMERIC: INTEGER h25

H INCOME AND EXPENDITURE

ADDITIONAL INCOME GENERATION

Is it a common practice to pursue non-health related income-generating activities, such as farming in addition to being a health professional?

SINGLE-SELECT h26

01 ○ Yes
02 ○ No

SINGLE-SELECT h27

How important do you think is the health-related income generated at the public health facility in comparison to other sources of income, such as farming or having a private practice for the household of the health professional?<br><br>E `h26==1`	01 ○ It is the main source of income<br>02 ○ Other income generating activities such as farming or private practice income are the main source of income<br>03 ○ They are equally important<br>97 ○ Others, specify
Specify other<br><br>E `h27==97`	TEXT h27oth<br>..

H INCOME AND EXPENDITURE

HOUSEHOLD FINANCE

Are you married or live with your partner?	SINGLE-SELECT h28<br>01 ○ Yes<br>02 ○ No
Is your spouse self-employed or earns a salary?<br><br>E `h28==1`	SINGLE-SELECT h30<br>01 ○ Self-employed<br>02 ○ Salary<br>03 ○ No, spouse does not work
How much money does your spouse make in a week?<br><br>I Enter value in thousand GNF 99998 I don't know<br>E `h30==1`<br>V1 `self.InRange(0,1250) || self==99998`	NUMERIC: INTEGER h31<br>_ _ _ _ _ _ _ _ _ _ _ _ _ _ _ _ _ _ _ _
How much money does your spouse make in a month?<br><br>I Enter value in thousand GNF 99998 I don't know<br>E `h30==2`<br>V1 `self.InRange(0,5000) || self==99998`	NUMERIC: INTEGER h32<br>_ _ _ _ _ _ _ _ _ _ _ _ _ _ _ _ _ _ _ _
How many other household members contribute to your household's monthly income?<br><br>V1 `self.InRange(0,21)`<br>M1 Out of Range	NUMERIC: INTEGER h29<br>_ _ _ _ _ _ _ _ _ _ _ _ _ _ _ _ _ _ _ _
How many financial dependents live in the household?<br><br>V1 `self.InRange(0,40)`	NUMERIC: INTEGER h34<br>_ _ _ _ _ _ _ _ _ _ _ _ _ _ _ _ _ _ _ _
What is your household's largest bulk expense?<br><br>I Definition life-cycle events. DO NOT PROMPT	SINGLE-SELECT h35<br>01 ○ School fees/uniforms<br>02 ○ Life cycle events<br>03 ○ Medicine/medical fees<br>04 ○ Agricultural inputs<br>05 ○ Food<br>06 ○ Rent<br>07 ○ Transport<br>98 ○ Don't want to respond<br>97 ○ Other, specify
Specify other<br><br>E `h35==97`	TEXT h35oth<br>..

How do you mainly pay for this bulk expense?<br><br>I DO NOT PROMPT	SINGLE-SELECT h36<br>01 ○ Loan: family/friends<br>02 ○ Loan: advance from work<br>03 ○ Savings<br>04 ○ Selling an asset<br>05 ○ Can cover with monthly salary<br>06 ○ Reduction of expenses<br>07 ○ Loan from financial institution<br>99 ○ Don't want to respond<br>97 ○ Others, specify
Specify other<br><br>E `h36==97`	TEXT h36oth<br>..
Does your household manage to meet these basic expenses every week?<br><br>I Do not prompt.	SINGLE-SELECT h37<br>01 ○ Yes, always<br>02 ○ Most of the time<br>03 ○ Rarely<br>04 ○ Never<br>99 ○ Prefer not to say

H INCOME AND EXPENDITURE

HOUSING AND TRANSPORTATION

How many days of annual leave do you have in a year?<br><br>I Write the number of days per year.<br>V1 `self.InRange(0,90)`<br>M1 Out of Range	NUMERIC: INTEGER h38<br>_ _ _ _ _ _ _ _ _ _ _ _ _ _ _ _ _ _ _ _
As part of your job, are you provided with housing?	SINGLE-SELECT h39<br>01 ○ Yes<br>02 ○ No
How do you rate the quality of the housing?<br><br>I Read out.<br>E `h39==1`	SINGLE-SELECT h40<br>01 ○ Excellent<br>02 ○ Good<br>03 ○ Decent<br>04 ○ Poor<br>05 ○ Terrible
In a normal week, how often do you sleep in the housing that has been provided?<br><br>I Write number of nights per week.<br>E `h39==1`<br>V1 `self.InRange(0,7)`<br>M1 Out of range	NUMERIC: INTEGER h41<br>_ _ _ _ _ _ _ _ _ _ _ _ _ _ _ _ _ _ _ _
Do you use a mode of transportation to carry out your job? Motorbike/car.	SINGLE-SELECT h42<br>01 ○ Yes<br>02 ○ No
Do you get sufficient fuel/ funds for fuel from your facility to use a motorbike for work purposes?<br><br>E `h42==1`	SINGLE-SELECT h43<br>01 ○ Yes, always<br>02 ○ Most of the time<br>03 ○ Rarely, or never

I ABSENTEEISM

Question	Response	Variable
On any given day, do you feel that many of your co-workers are absent from work in this facility?<br><br>I Many: more than 1/4.	SINGLE-SELECT<br>01 ○ Yes<br>02 ○ No<br>99 ○ Prefer not to say	i1
In any given month, on average, how many days do you think your co-workers are absent in this facility?<br><br>I 99998 Don't know<br>V1 `self.InRange(0,30) || self==99998`<br>M1 Out of Range	NUMERIC: INTEGER<br>_ _ _ _ _ _ _ _ _ _	i2
What would you say is the main reason?<br><br>I DO NOT PROMPT	SINGLE-SELECT<br>00001 ○ Travelling to work takes too long<br>00002 ○ Sickness<br>00003 ○ Sick relatives/family<br>00004 ○ Other jobs (non-health related)<br>00005 ○ Private practice<br>00006 ○ Family related reasons<br>00007 ○ Household obligations<br>00008 ○ Tired from previous days<br>00097 ○ Others, specify<br>99998 ○ I don't know	i3
Specify other<br><br>E `i3==97`	TEXT<br>....................................	i3oth
Do you feel as if you worked more days or less days than your co-workers?	SINGLE-SELECT<br>01 ○ More<br>02 ○ Same<br>03 ○ Less<br>99 ○ Not applicable	i4
In the last 30 days, were you absent from work because you were sick? How many days?<br><br>I Enter number of days, enter 0 if 'No'<br>V1 `self.InRange(0,30)`<br>M1 Out of Range	NUMERIC: INTEGER<br>_ _ _ _ _ _ _ _ _ _	i5
In the last 30 days, were you absent from work because of trainings, outreach and authorized leave? How many days?<br><br>I Enter number of days, enter 0 if 'No'<br>V1 `self.InRange(0,30)`<br>M1 Out of Range	NUMERIC: INTEGER<br>_ _ _ _ _ _ _ _ _ _	i6
In the last 30 days, how many days were you absent from work because of other, personal reasons?<br><br>I Enter number of days, enter 0 if 'No'<br>V1 `self.InRange(0,30)`<br>M1 Out of Range	NUMERIC: INTEGER<br>_ _ _ _ _ _ _ _ _ _	i7
What was the reason for your absence from work last time when you were away from work for personal reasons?<br><br>I Do not prompt.	SINGLE-SELECT<br>01 ○ Travelling to work took too long<br>02 ○ I was sick<br>03 ○ Sick relatives/family	i8

	04 I have another job to attend<br>05 Private practice<br>06 I must care for children<br>07 I had household chores<br>08 I was tired from previous day<br>99 Never been away from work for personal reasons<br>97 Others, specify
Specify other<br><br>E i8==97	TEXT i8oth<br><br>..

J SATISFACTION AND CONSTRAINTS

J SATISFACTION AND CONSTRAINTS

SATISFACTION

How satisfied are you with life in general?<br><br>I Read out answer options	SINGLE-SELECT j1a<br>01 Very satisfied<br>02 Satisfied<br>03 Unsatisfied<br>04 Very unsatisfied
How satisfied are you with the financial situation of your life?<br><br>I Read out answer options	SINGLE-SELECT j1b<br>01 Very satisfied<br>02 Satisfied<br>03 Unsatisfied<br>04 Very unsatisfied
How satisfied are you with the working conditions?	SINGLE-SELECT j1d<br>01 Very satisfied<br>02 Satisfied<br>03 Unsatisfied<br>04 Very unsatisfied
How satisfied are you with the balance between work and leisure time of your life?	SINGLE-SELECT j1c<br>01 Very satisfied<br>02 Satisfied<br>03 Unsatisfied<br>04 Very unsatisfied
How satisfied are you with the career opportunities of your life?	SINGLE-SELECT j1e<br>01 Very satisfied<br>02 Satisfied<br>03 Unsatisfied<br>04 Very unsatisfied

J SATISFACTION AND CONSTRAINTS

MOTIVATIONAL STATEMENTS

How much do you agree with the followin statements? I have enough opportunities to learn at the health facility.<br><br>I Read out answer options	SINGLE-SELECT j2a<br>01 Strongly agree<br>02 Agree<br>03 Disagree<br>04 Strongly disagree
Career prospectives are fair and promotions are based on performance.	SINGLE-SELECT j2b<br>01 Strongly agree

	02 ○ Agree<br>03 ○ Disagree<br>04 ○ Strongly disagree
I feel motivated to go to work every day.	SINGLE-SELECT j2c<br>01 ○ Strongly agree<br>02 ○ Agree<br>03 ○ Disagree<br>04 ○ Strongly disagree
My salary is too low to meet the basic expenses of my family.	SINGLE-SELECT j2d<br>01 ○ Strongly agree<br>02 ○ Agree<br>03 ○ Disagree<br>04 ○ Strongly disagree
Health workers have to ask for payments from patients to make a living.	SINGLE-SELECT j2e<br>01 ○ Strongly agree<br>02 ○ Agree<br>03 ○ Disagree<br>04 ○ Strongly disagree
I worry about being unemployed.	SINGLE-SELECT j2f<br>01 ○ Strongly agree<br>02 ○ Agree<br>03 ○ Disagree<br>04 ○ Strongly disagree
We have sufficient drugs and supplies to treat patients.	SINGLE-SELECT j2h<br>01 ○ Strongly agree<br>02 ○ Agree<br>03 ○ Disagree<br>04 ○ Strongly disagree
I worry about being moved to another area.	SINGLE-SELECT j2g<br>01 ○ Strongly agree<br>02 ○ Agree<br>03 ○ Disagree<br>04 ○ Strongly disagree
We have enough equipment in working condition to treat the patients.	SINGLE-SELECT j2i<br>01 ○ Strongly agree<br>02 ○ Agree<br>03 ○ Disagree<br>04 ○ Strongly disagree
When I do my job well, my opinion of myself improves.	SINGLE-SELECT j2j<br>01 ○ Strongly agree<br>02 ○ Agree<br>03 ○ Disagree<br>04 ○ Strongly disagree
I work here because the salary reflects each health professional's contribution fairly.	SINGLE-SELECT j2k<br>01 ○ Strongly agree<br>02 ○ Agree<br>03 ○ Disagree<br>04 ○ Strongly disagree
It is important for me to have opportunities to advance my career.	SINGLE-SELECT j2l<br>01 ○ Strongly agree<br>02 ○ Agree<br>03 ○ Disagree<br>04 ○ Strongly disagree

Question	Response	Code
I am glad I work in this facility rather than in any other health facility.	SINGLE-SELECT<br>01 ○ Strongly agree<br>02 ○ Agree<br>03 ○ Disagree<br>04 ○ Strongly disagree	j2m
I feel personally responsible if we cannot meet the needs of a patient.	SINGLE-SELECT<br>01 ○ Strongly agree<br>02 ○ Agree<br>03 ○ Disagree<br>04 ○ Strongly disagree	j2n
I frequently think of quitting this job.	SINGLE-SELECT<br>01 ○ Strongly agree<br>02 ○ Agree<br>03 ○ Disagree<br>04 ○ Strongly disagree	j2o
My colleagues are supportive, that’s why I like working here.	SINGLE-SELECT<br>01 ○ Strongly agree<br>02 ○ Agree<br>03 ○ Disagree<br>04 ○ Strongly disagree	j2p
I work here because it provides long term security for me.	SINGLE-SELECT<br>01 ○ Strongly agree<br>02 ○ Agree<br>03 ○ Disagree<br>04 ○ Strongly disagree	j2q
I work here because of opportunities for promotion.	SINGLE-SELECT<br>01 ○ Strongly agree<br>02 ○ Agree<br>03 ○ Disagree<br>04 ○ Strongly disagree	j2r
I feel like I can accomplish something worthwhile in this job.	SINGLE-SELECT<br>01 ○ Strongly agree<br>02 ○ Agree<br>03 ○ Disagree<br>04 ○ Strongly disagree	j2s
I work here because I enjoy doing this job.	SINGLE-SELECT<br>01 ○ Strongly agree<br>02 ○ Agree<br>03 ○ Disagree<br>04 ○ Strongly disagree	j2t
It’s difficult to care very much whether the work gets done right.	SINGLE-SELECT<br>01 ○ Strongly agree<br>02 ○ Agree<br>03 ○ Disagree<br>04 ○ Strongly disagree	j2u
I sometimes feel that my work is meaningless.	SINGLE-SELECT<br>01 ○ Strongly agree<br>02 ○ Agree<br>03 ○ Disagree<br>04 ○ Strongly disagree	j2v
It is important for me to get recognition from the community for my work.	SINGLE-SELECT<br>01 ○ Strongly agree<br>02 ○ Agree	j2w

	03 ○ Disagree<br>04 ○ Strongly disagree

K CHARACTERISTICS OF THE RESPONDENT

Question	Response
What is your gender?	SINGLE-SELECT k1<br>01 ○ Male<br>02 ○ Female
What is your age?<br>I Enter age in completed years<br>V1 `self.InRange(0,80)`<br>M1 Out of Range	NUMERIC: INTEGER k2<br>_ _
What is your marital status?	SINGLE-SELECT k3<br>01 ○ Single<br>02 ○ Engaged<br>03 ○ In a living relation<br>04 ○ Married<br>05 ○ Widowed<br>06 ○ Divorced<br>07 ○ Separated
Is your spouse/ partner working or studying in the health sector?<br>E `k3.InList(2,3,4)`	SINGLE-SELECT k4<br>01 ○ Yes<br>02 ○ No
Where does your spouse/partner live?<br>E `k3.InList(2,3,4)`	SINGLE-SELECT k5<br>01 ○ Boké<br>02 ○ Conakry<br>03 ○ Faranah<br>04 ○ Kankan<br>05 ○ Kindia<br>06 ○ Labé<br>07 ○ Mamou<br>08 ○ Nzérékoré<br>09 ○ Abroad
Where?<br>E `k3 != 1 && k5==9`	TEXT k5oth<br>..
How many children do you have?<br>I Write 0 if none.	NUMERIC: INTEGER k6<br>_ _
In which region were you born?	SINGLE-SELECT k7<br>01 ○ Boké<br>02 ○ Conakry<br>03 ○ Faranah<br>04 ○ Kankan<br>05 ○ Kindia<br>06 ○ Labé<br>07 ○ Mamou<br>08 ○ Nzérékoré<br>09 ○ Abroad
Where?<br>E `k7==9`	TEXT k7oth<br>..

In which region did you grow up?	SINGLE-SELECT k8<br>01 ○ Boké<br>02 ○ Conakry<br>03 ○ Faranah<br>04 ○ Kankan<br>05 ○ Kindia<br>06 ○ Labé<br>07 ○ Mamou<br>08 ○ Nzérékoré<br>09 ○ Abroad
Where?<br>E k8==9	TEXT k8oth<br>..
What was/is your father's highest level of education?	SINGLE-SELECT k13<br>01 ○ No education<br>03 ○ Completed primary education<br>04 ○ Completed secondary education<br>05 ○ Completed technical education or vocational training<br>06 ○ University education<br>99 ○ Don't Know<br>97 ○ Others, specify
Specify other<br>E k13==97	TEXT k13oth<br>..
What was/is your father's main field of occupation?	SINGLE-SELECT k14<br>01 ○ Work in the house<br>02 ○ Tailor/ dressmaker<br>03 ○ Trader / merchant<br>04 ○ Student / pupil<br>05 ○ Worker (bricklayer, mechanic, carpenter, electrician, painter)<br>06 ○ Farmer<br>07 ○ Driver<br>08 ○ Stockbreeder<br>09 ○ Sinner<br>10 ○ Employee / agronomist / engineer / teacher<br>11 ○ Health professional<br>97 ○ Others, specify
Specify other<br>E k14==97	TEXT k14oth<br>..
Who owned the dwelling in which you grew up in?	SINGLE-SELECT k15<br>01 ○ Household<br>02 ○ Relations of head of household<br>03 ○ State<br>04 ○ Company or private enterprise<br>05 ○ Private landlord<br>97 ○ Others, specify
Specify other<br>E k15==97	TEXT k15oth<br>..
How would you classify the socio-economic background of your parents?	SINGLE-SELECT k12<br>01 ○ Rich

	02 ○ Upper middle class<br>03 ○ Lower middle class<br>04 ○ Poor<br>05 ○ Very poor
Would you classify the house that you grew up in as being in an urban area or a rural area?	SINGLE-SELECT k16<br>01 ○ Urban<br>02 ○ Rural
Approximately, how far away was the closest all weather road from the house you grew up in, in kilometres?<br>I Write in approximate kilometres<br>V1 `self.InRange(0,300)`<br>M1 Out of Range	NUMERIC: INTEGER k17<br>_ _
Did the household you grew up in have any of the following items?	MULTI-SELECT: YES/NO k18<br>02 ☐ / ☐ Refrigerator<br>03 ☐ / ☐ Radio<br>04 ☐ / ☐ Cassette/ CD Player<br>05 ☐ / ☐ Television<br>06 ☐ / ☐ Running Water<br>07 ☐ / ☐ Electricity<br>08 ☐ / ☐ Telephone<br>09 ☐ / ☐ Car<br>10 ☐ / ☐ Motorcycle<br>11 ☐ / ☐ Bicycle<br>12 ☐ / ☐ Own Land<br>13 ☐ / ☐ Own House
Does your household own any of the following?	MULTI-SELECT: YES/NO k9<br>02 ☐ / ☐ Refrigerator<br>03 ☐ / ☐ Radio<br>04 ☐ / ☐ Cassette/ CD Player<br>05 ☐ / ☐ Television<br>06 ☐ / ☐ Running Water<br>07 ☐ / ☐ Electricity<br>08 ☐ / ☐ Telephone<br>09 ☐ / ☐ Car<br>10 ☐ / ☐ Motorcycle<br>11 ☐ / ☐ Bicycle<br>12 ☐ / ☐ Own Land<br>13 ☐ / ☐ Own House
How would you classify your household's socio-economic profile?	SINGLE-SELECT k12a<br>01 ○ Rich<br>02 ○ Upper middle class<br>03 ○ Lower middle class<br>04 ○ Poor<br>05 ○ Very poor
Have you ever been to any other country in Africa?	SINGLE-SELECT k10<br>01 ○ Yes<br>02 ○ No
Have you ever been to any other country outside Africa?	SINGLE-SELECT k11<br>01 ○ Yes<br>02 ○ No

L END OF INTERVIEW

Interview end time	DATE: CURRENT TIME<br>..	l1
Interview result	SINGLE-SELECT<br>01 ○ Completed<br>02 ○ Incomplete<br>97 ○ Others, specify	l2
Interview result<br>E l2==97	TEXT<br>..	l2_oth
Interviewer’s comments	TEXT<br>..	l3

LEGEND

Legend and structure of information in this file

Name of section

Enabling condition for this section

Question title

Type of question, scope

Variable name

Answer options

SECTION 5: OTHER INCOME SOURCES

E s4_other_sources_which.Contains(98)

Duis aute irure dolor in reprehenderit in voluptate velit esse cillum dolore eu fugiat nulla pariatur?

I This refers to family relations
E s3_time_other > 0
V1 s4_rel_leaders_which.Contains(98)
M1 Can not be itself
V2 (s3_time_other_breeding_advice <= (50 - s3_time_art_insem_advice)) ||s3_time_other_breeding_advice == 0
M2 This person is not in the list
F optioncode != s5_ignored_option_code

MULTI-SELECT
SCOPE: PREFILLED

s4_rel_leaders_other

01 ☐ Community animal health workers
02 ☐ Private
03 ☐ Government
04 ☐ Livestock keepers association
05 ☐ NGO

And 5 other [13]

Additional information:
"I" — Question instruction
"E" — Enabling condition
"V1" — Validation condition №1
"M1" — Message for validation №1
"F" — Filter in Categorical questions

Link to full set in appendix

Breadcrumbs

Type or roster

Roster Title

CHAPTER 3 IDENTIFICATION /

Roster: **LEADER RELATION DETAILS**

generated by fixed list:

01 Ward Livestock Officer
02 Village Livestock Officer
99 Other (specify)

List items
