# Supplementary material for: How to attract and retain health workers in rural areas of a fragile state: Findings from a labour market survey in Guinea
Source: PLoS One. 2021 Dec 16;16(12):e0245569. doi: 10.1371/journal.pone.0245569 (PMC8675729; doi:10.1371/journal.pone.0245569)
Supplement: S2 File — (PDF) [file pone.0245569.s002.pdf]

This PDF generated by hlaufer, 1/29/2018 5:48:13 AM
Questionnaire created by andreaskutka, 11/20/2017 9:32:43 PM
Last modified by andreaskutka, 11/22/2017 4:53:57 PM

Not shared with anyone

Sections: 12, Sub-sections: 12,
Questions: 241.
Questions with enabling conditions: 65
Questions with validation conditions:45
Rosters: 0
Variables: 1

Professionnels de santé

A ADMINISTRATION
No sub-sections, No rosters, Questions: 13, Variables: 1.

B CONSENTEMENT
No sub-sections, No rosters, Questions: 6, Static texts: 1.

C IDENTIFICATION
No sub-sections, No rosters, Questions: 3.

D CHOIX DU TRAVAIL
No sub-sections, No rosters, Questions: 35, Static texts: 21.

E TRAVAIL ACTUEL
Sub-sections: 5, No rosters, Questions: 36.

F PRÉFÉRENCES
No sub-sections, No rosters, Questions: 10.

G ANTÉCÉDENTS PROFESSIONNELS
No sub-sections, No rosters, Questions: 25, Static texts: 3.

H REVENU ET DÉPENSES
Sub-sections: 5, No rosters, Questions: 46, Static texts: 1.

I ABSENTÉISME
No sub-sections, No rosters, Questions: 10.

J SATISFACTION ET CONTRAINTES
Sub-sections: 2, No rosters, Questions: 28.

K DESCRIPTION DE LA PERSONNE INTERROGÉE
No sub-sections, No rosters, Questions: 25.

L FIN DE L’ENTRETIEN
No sub-sections, No rosters, Questions: 4.

LEGEND

A ADMINISTRATION

Code ID de l'établissement	TEXT<br>SCOPE: IDENTIFYING<br>..	hfid
Code ID de la personne réalisant l'entretien	TEXT<br>..	a1
Code ID du (de la) responsable	TEXT<br>..	a2
Date de l'entretien	DATE: CURRENT TIME<br>..	a3
Nom de l'établissement	TEXT<br>..	a4
Type d'établissement de santé	SINGLE-SELECT<br>01 ○ Hôpital national<br>02 ○ Hôpital régional<br>03 ○ Hôpital préfectoral<br>04 ○ Centre médico-communal<br>05 ○ Centre de santé améliore<br>06 ○ Centre de santé<br>07 ○ Poste de santé	a5
Lieu	SINGLE-SELECT<br>01 ○ Urbain<br>02 ○ Rural	a6
Zone	SINGLE-SELECT<br>01 ○ Zone 1<br>02 ○ Zone 2<br>03 ○ Zone 3<br>04 ○ Zone 4<br>98 ○ Je ne sais pas	a7
Région	SINGLE-SELECT<br>01 ○ Boké<br>02 ○ Conakry<br>03 ○ Faranah<br>04 ○ Kankan<br>05 ○ Kindia<br>06 ○ Labé<br>07 ○ Mamou<br>08 ○ Nzérékoré	a8
Préfecture	TEXT<br>..	a9
Sous-Préfecture	TEXT<br>..	a10
Ville la plus proche	TEXT<br>..	a11

Coordonnées GPS	GPS a12<br>N ____<br>W ____<br>A ____
VARIABLE<br>Quest.IRnd()	DOUBLE rnd

B CONSENTEMENT

STATIC TEXT

Bonjour. Je m'appelle ________________________ et je viens de CERREGUI. Nous travaillons avec le Ministère de la santé, la Banque mondiale et Oxford Policy Management sur ce projet d'analyse de la santé. Nous aimerions savoir comment vous êtes devenu un professionnel de santé, à quels défis vous devez faire face dans votre travail quotidien, quelles sont vos motivations et vos préférences. Vos réponses nous aideront à mieux comprendre votre situation en tant qu'agent de santé. Nous présenterons les résultats au Ministère de la santé pour permettre aux acteurs politiques de concevoir des améliorations dans les conditions de travail pour les professionnels de santé en Guinée. L'entretien durera environ 60 minutes. La participation à cette étude est entièrement volontaire. Les conclusions seront rapportées de telle sorte que personne ne saura ce que vous me direz aujourd'hui. Les résultats seront groupés, sans afficher de détails sur les participants. Cela signifie que vous pouvez être totalement honnête avec nous. Bien entendu, vous pouvez refuser de répondre à toute question et arrêter l'entretien à tout moment si vous le souhaitez. Avez-vous déjà des questions au sujet de l'étude ? Ai-je votre accord pour continuer dans cette voie ?

Je comprends que les renseignements que je fournirai pour les biens de l'enquête incluent des identificateurs qui permettront de me contacter pour d'éventuels éclaircissements. Je comprends que les informations relatives à l'identificateur ne seront pas utilisées dans l'analyse. Chaque formulaire sera rendu anonyme et aucune information personnelle ne sera partagée avec quiconque autre que l'équipe de recherche.<br>V1 self==1<br>M1 EXPLIQUER AU RÉPONDANT DE FAÇON SIMPLE	SINGLE-SELECT b1<br>01 ○ Oui<br>02 ○ Non
Je comprends que ma participation est volontaire et que je suis libre de me retirer à tout moment, sans en donner la raison, et sans que mon emploi, ou mes droits juridiques soient affectés.<br>V1 self==1<br>M1 EXPLIQUER AU RÉPONDANT DE FAÇON SIMPLE	SINGLE-SELECT b2<br>01 ○ Oui<br>02 ○ Non
Je comprends que les informations de mon entretien seront mises en commun avec les réponses d'autres participants, et qu'elles seront rendues anonymes et qu'elles peuvent être publiées.<br>V1 self==1<br>M1 EXPLIQUER AU RÉPONDANT DE FAÇON SIMPLE	SINGLE-SELECT b3<br>01 ○ Oui<br>02 ○ Non
Souhaitez-vous participer à notre enquête ?<br>E b1==1 && b2==1 && b3==1	SINGLE-SELECT b4<br>01 ○ Oui<br>02 ○ Non
Si vous ne souhaitez pas participer à cette étude, pourriez-vous nous indiquer votre raison principale ?	SINGLE-SELECT b5<br>01 ○ Je n'ai pas le temps

E b4==2	02 ○ Ça ne m'intéresse pas<br>03 ○ Les supérieurs ne l'acceptent pas<br>04 ○ Il s'agit de renseignements personnels<br>05 ○ Peur/ anxiété<br>97 ○ Autre, précisez<br>98 ○ Je ne sais pas
Précisez autre<br>E b5==97	TEXT b5_oth<br>..

C IDENTIFICATION

Nom et prénom de la personne	TEXT c1<br>..
Quelle est votre profession ?	SINGLE-SELECT c2<br>01 ○ Médecin spécialiste<br>02 ○ Médecin<br>03 ○ Infirmier (ère)<br>04 ○ Sage-femme<br>05 ○ ATS
Numéro de téléphone portable<br>I Écrire 000000000 s'il n'y en a pas	TEXT c3<br>..

D CHOIX DU TRAVAIL

STATIC TEXT

À lire par la personne réalisant l'entretien : Dans cette partie du questionnaire, nous voulons essayer de comprendre les facteurs qui agissent sur les préférences professionnelles des médecins. Il y a de nombreuses caractéristiques importantes pour un emploi, mais nous avons choisi de nous concentrer sur sept d'entre elles : le lieu, le type d'établissement, le matériel de l'établissement, le logement, le transport, le revenu et la formation. Nous vous présenterons 16 séries de description de postes de travail hypothétiques. Dans chaque série, les postes sont appelés « Poste A » et « Poste B ». Les caractéristiques du « Poste A » et du « Poste B » varient selon les séries. Chaque emploi présente des avantages et des inconvénients et vous devrez évaluer ces avantages et inconvénients et faire un compromis en choisissant quel emploi vous préférez.

STATIC TEXT

A MONTRER:
(1) la feuille avec les differentes characteristiques en rapport avec le type de travailleur: %c2%
(2) L'exemple pour le travailleur %c2%

STATIC TEXT

E (c2==1 || c2 == 2) && rnd<0.5

Procédure alternative: Veuillez passer la tablette à l'enqueté(e) et faire exemple avec lui/elle

Médecins: EXEMPLE

Caractéristiques	Poste A	Poste B
Salaire	6.5 millions	2 millions
Localisation	Conakry	capitale préfectorale
Equipement	Bon	Mediocre
Formation	spécialisation	aucun
Temps d'affectation	indéterminée	indéterminée
Logement	logement	aucun
Transport	moto	aucun

Exemple: faites le choix	SINGLE-SELECT `choice_ex`
E `(c2==1 || c2 == 2) && rnd<0.5`	01 ○ Job A<br>02 ○ Job B

STATIC TEXT

E `rnd>=0.5 || c2==3 || c2 == 4 || c2 == 5`

Procédure standard: Veuillez montrer et lire les cartes a la personne enquêtée

Mettre l'heure de depart	DATE: CURRENT TIME `DCE_start_time`<br>..

STATIC TEXT

E `(c2==1 || c2 == 2) && rnd<0.5`

Choix Numéro 1

Caractéristiques	Poste A	Poste B
Salaire	6.5 millions	6.5 millions
Localisation	capitale préfectorale	Conakry
Equipement	Médiocre	bon
Formation	spécialisation	ateliers
Temps d'affectation	indéterminée	indéterminée
Logement	aucun	aucun
Transport	moto	aucun

Choix numéro 1	SINGLE-SELECT `choice1`
	01 ○ Job A<br>02 ○ Job B
Si vous pouviez avoir ce poste, l'accepteriez-vous?	SINGLE-SELECT `choice1_would`<br>01 ○ oui<br>02 ○ non

STATIC TEXT

E `(c2==1 || c2 == 2) && rnd<0.5`

Choix Numéro 2

Caractéristiques	Poste A	Poste B
Salaire	3.5 millions	2 millions
Localisation	capitale préfectorale	capitale sous-préfectorale (CS)
Equipement	moyen	médiocre
Formation	ateliers	ateliers
Temps d'affectation	indéterminée	5 ans
Logement	aucun	logement
Transport	moto	aucun

Choix numéro 2	SINGLE-SELECT `choice2`
	01 ○ Job A<br>02 ○ Job B

Si vous pouviez avoir ce poste, l'accepteriez-vous?

SINGLE-SELECT — choice2_would

01 oui
02 non

STATIC TEXT

E (c2==1 || c2 == 2) && rnd<0.5

Choix Numéro 3

Caractéristiques	Poste A	Poste B
Salaire	2 millions	3.5 millions
Localisation	Conakry	capitale préfectorale
Equipement	moyen	moyen
Formation	spécialisation	ateliers
Temps d'affectation	indéterminée	indéterminée
Logement	aucun	logement
Transport	aucun	moto

Choix numéro 3

SINGLE-SELECT — choice3

01 Job A
02 Job B

Si vous pouviez avoir ce poste, l'accepteriez-vous?

SINGLE-SELECT — choice3_would

01 oui
02 non

STATIC TEXT

E (c2==1 || c2 == 2) && rnd<0.5

Choix Numéro 4

Caractéristiques	Poste A	Poste B
Salaire	2 millions	6.5 millions
Localisation	capitale préfectorale	capitale sous-préfectorale (CS)
Equipement	bon	moyen
Formation	aucun	aucun
Temps d'affectation	indéterminée	5 ans
Logement	logement	aucun
Transport	aucun	moto

Choix numéro 4

SINGLE-SELECT — choice4

01 Job A
02 Job B

Si vous pouviez avoir ce poste, l'accepteriez-vous?

SINGLE-SELECT — choice4_would

01 oui
02 non

STATIC TEXT

E (c2==1 || c2 == 2) && rnd<0.5

Choix Numéro 5

Caractéristiques	Poste A	Poste B
Salaire	3.5 millions	2 millions
Localisation	Conakry	capitale sous-préfectorale (CS)
Equipement	médiocre	médiocre
Formation	aucun	ateliers
Temps d'affectation	indéterminée	indéterminée
Logement	aucun	aucun
Transport	aucun	moto

Choix numéro 5

SINGLE-SELECT — choice5

	01 ○ Job A<br>02 ○ Job B
Si vous pouviez avoir ce poste, l'accepteriez-vous?	SINGLE-SELECT choice5_would<br>01 ○ oui<br>02 ○ non

STATIC TEXT

E (c2==1 || c2 == 2) && rnd<0.5

Choix Numéro 6

Caractéristiques	Poste A	Poste B
Salaire	2 millions	2 millions
Localisation	Conakry	capitale préfectorale
Equipement	moyen	bon
Formation	spécialisation	aucun
Temps d'affectation	indéterminée	indéterminée
Logement	aucun	aucun
Transport	aucun	moto

Choix numéro 6	SINGLE-SELECT choice6<br>01 ○ Job A<br>02 ○ Job B
Si vous pouviez avoir ce poste, l'accepteriez-vous?	SINGLE-SELECT choice6_would<br>01 ○ oui<br>02 ○ non

STATIC TEXT

E (c2==1 || c2 == 2) && rnd<0.5

Choix Numéro 7

Caractéristiques	Poste A	Poste B
Salaire	3.5 millions	3.5 millions
Localisation	capitale préfectorale	capitale sous-préfectorale (CS)
Equipement	moyen	bon
Formation	ateliers	spécialisation
Temps d'affectation	indéterminée	indéterminée
Logement	aucun	logement
Transport	moto	aucun

Choix numéro 7	SINGLE-SELECT choice7<br>01 ○ Job A<br>02 ○ Job B
Si vous pouviez avoir ce poste, l'accepteriez-vous?	SINGLE-SELECT choice7_would<br>01 ○ oui<br>02 ○ non

STATIC TEXT

E (c2==1 || c2 == 2) && rnd<0.5

Choix Numéro 8

Caractéristiques	Poste A	Poste B
Salaire	3.5 millions	2 millions
Localisation	capitale sous-préfectorale (CS)	capitale préfectorale
Equipement	bon	bon
Formation	spécialisation	aucun
Temps d'affectation	indéterminée	5 ans
Logement	aucun	logement
Transport	aucun	moto

Choix numéro 8	SINGLE-SELECT choice8
	01 ○ Job A<br>02 ○ Job B

Si vous pouviez avoir ce poste, l'accepteriez-vous?	SINGLE-SELECT choice8_would
	01 ○ oui<br>02 ○ non

STATIC TEXT

E (c2==1 || c2 == 2) && rnd<0.5

Choix Numéro 9

Caractéristiques	Poste A	Poste B
Salaire	6.5 millions	2 millions
Localisation	capitale sous-préfectorale (CS)	Conakry
Equipement	moyen	moyen
Formation	aucun	spécialisation
Temps d'affectation	5 ans	indéterminée
Logement	aucun	aucun
Transport	aucun	aucun

Choix numéro 9	SINGLE-SELECT choice9
	01 ○ Job A<br>02 ○ Job B

Si vous pouviez avoir ce poste, l'accepteriez-vous?	SINGLE-SELECT choice9_would
	01 ○ oui<br>02 ○ non

STATIC TEXT

E (c2==1 || c2 == 2) && rnd<0.5

Choix Numéro 10

Caractéristiques	Poste A	Poste B
Salaire	6.5 millions	3.5 millions
Localisation	Conakry	capitale préfectorale
Equipement	bon	moyen
Formation	ateliers	ateliers
Temps d'affectation	indéterminée	5 ans
Logement	aucun	logement
Transport	aucun	aucun

Choix numéro 10	SINGLE-SELECT choice10
	01 ○ Job A<br>02 ○ Job B

Si vous pouviez avoir ce poste, l'accepteriez-vous?	SINGLE-SELECT choice10_would
	01 ○ oui<br>02 ○ non

STATIC TEXT

E (c2==1 || c2 == 2) && rnd<0.5

Choix Numéro 11

Caractéristiques	Poste A	Poste B
Salaire	3.5 millions	3.5 millions
Localisation	capitale sous-préfectorale (CS)	Conakry
Equipement	bon	médiocre
Formation	spécialisation	aucun
Temps d'affectation	indéterminée	indéterminée
Logement	aucun	aucun
Transport	aucun	aucun

Choix numéro 11	SINGLE-SELECT<br>01 ○ Job A<br>02 ○ Job B	choice11
Si vous pouviez avoir ce poste, l'accepteriez-vous?	SINGLE-SELECT<br>01 ○ oui<br>02 ○ non	choice11_would

STATIC TEXT

E (c2==1 || c2 == 2) && rnd<0.5

Choix Numéro 12

Caractéristiques	Poste A	Poste B
Salaire	3.5 millions	6.5 millions
Localisation	Conakry	capitale sous-préfectorale (CS)
Equipement	médiocre	moyen
Formation	aucun	aucun
Temps d'affectation	indéterminée	5 ans
Logement	aucun	logement
Transport	aucun	aucun

Choix numéro 12	SINGLE-SELECT<br>01 ○ Job A<br>02 ○ Job B	choice12
Si vous pouviez avoir ce poste, l'accepteriez-vous?	SINGLE-SELECT<br>01 ○ oui<br>02 ○ non	choice12_would

STATIC TEXT

E (c2==1 || c2 == 2) && rnd<0.5

Choix Numéro 13

Caractéristiques	Poste A	Poste B
Salaire	2 millions	2 millions
Localisation	capitale sous-préfectorale (CS)	capitale préfectorale
Equipement	médiocre	bon
Formation	ateliers	aucun
Temps d'affectation	indéterminée	5 ans
Logement	logement	aucun
Transport	moto	aucun

Choix numéro 13	SINGLE-SELECT<br>01 ○ Job A<br>02 ○ Job B	choice13
Si vous pouviez avoir ce poste, l'accepteriez-vous?	SINGLE-SELECT<br>01 ○ oui	choice13_would

	02 ○ non

STATIC TEXT

E `(c2==1 || c2 == 2) && rnd<0.5`

Choix Numéro 14

Caractéristiques	Poste A	Poste B
Salaire	6.5 millions	3.5 millions
Localisation	capitale sous-préfectorale (CS)	capitale préfectorale
Equipement	moyen	moyen
Formation	aucun	ateliers
Temps d'affectation	indéterminée	5 ans
Logement	logement	aucun
Transport	moto	aucun

Choix numéro 14	SINGLE-SELECT `choice14`<br>01 ○ Job A<br>02 ○ Job B
Si vous pouviez avoir ce poste, l'accepteriez-vous?	SINGLE-SELECT `choice14_would`<br>01 ○ oui<br>02 ○ non

STATIC TEXT

E `(c2==1 || c2 == 2) && rnd<0.5`

Choix Numéro 15

Caractéristiques	Poste A	Poste B
Salaire	6.5 millions	6.5 millions
Localisation	capitale préfectorale	capitale sous-préfectorale (CS)
Equipement	médiocre	moyen
Formation	spécialisation	aucun
Temps d'affectation	5 ans	indéterminée
Logement	aucun	logement
Transport	moto	aucun

Choix numéro 15	SINGLE-SELECT `choice15`<br>01 ○ Job A<br>02 ○ Job B
Si vous pouviez avoir ce poste, l'accepteriez-vous?	SINGLE-SELECT `choice15_would`<br>01 ○ oui<br>02 ○ non

STATIC TEXT

E `(c2==1 || c2 == 2) && rnd<0.5`

Choix Numéro 16

Caractéristiques	Poste A	Poste B
Salaire	6.5 millions	3.5 millions
Localisation	capitale préfectorale	capitale sous-préfectorale (CS)
Equipement	médiocre	bon
Formation	spécialisation	spécialisation
Temps d'affectation	indéterminée	5 ans
Logement	logement	aucun
Transport	aucun	moto

Choix numéro 16	SINGLE-SELECT `choice16`<br>01 ○ Job A<br>02 ○ Job B

Si vous pouviez avoir ce poste, l'accepteriez-vous?

SINGLE-SELECT choice16_would

01 oui
02 non

STATIC TEXT

E (c2==1 || c2 == 2) && rnd<0.5

Merci ! Veuillez rendre la tablette a l'enquêteur

Mettre l'heure de fin

DATE: CURRENT TIME DCE_end_time

E TRAVAIL ACTUEL

E TRAVAIL ACTUEL

POSTE ACTUEL

Depuis combien d’années travaillez-vous à votre poste actuel ? (dans cet établissement de santé)

I Écrire en années complètes. Si c'est moins d'un an, écrire '0'
V1 self.InRange(0,70)
M1 Il devrait être entre 0 à 70. S.v.p. confirmer et entrer à nouveau.

NUMERIC: INTEGER e4

Avez-vous choisi de travailler dans cet établissement ?

SINGLE-SELECT e5

01 Oui
02 Non

Depuis combien d’années travaillez-vous dans cet établissement ?

I Écrire en années complètes. Si c'est moins d'un an, écrire '0'
V1 self.InRange(0,70)
M1 Il devrait être entre 0 à 70. S.v.p. confirmer et entrer à nouveau.
V2 self>=e4
M2 Ne put pas être inférieur au nombre d'années au poste actuel

NUMERIC: INTEGER e1

Dans combien de postes différents avez-vous travaillé dans cet établissement de santé?

I Écrire le nombre de postes, écrire '1' si ce n'est qu'un seul poste
V1 self.InRange(1,20)

NUMERIC: INTEGER e1_1

Avez-vous un poste permanent ou contractuel ?

I NE SUGGÉREZ PAS DE RÉPONSE

SINGLE-SELECT e2

01 Fonction publique (titulaire)
02 Contractuel payé par communauté
03 Contractuel payé par l'établissement de santé
04 Contractuel payé par les institutions de coopération

Quel est votre fonction ?

SINGLE-SELECT e3

01 Gestionnaire
02 Fonction médicale
03 Fonction soignant
04 Plusieurs
97 Autre, précisez

Specify other

TEXT e3_oth

E e3==97	....
Avez-vous recu(e) votre formation de base dans cette région ?	SINGLE-SELECT e6<br>01 ○ Oui<br>02 ○ Non
Où avez-vous recu(e) votre formation de base ?<br>E e6==2	SINGLE-SELECT e7<br>01 ○ Boké<br>02 ○ Conakry<br>03 ○ Faranah<br>04 ○ Kankan<br>05 ○ Kindia<br>06 ○ Labé<br>07 ○ Mamou<br>08 ○ Nzérékoré
Avez-vous travaillé en milieu rural durant votre formation de base ?	SINGLE-SELECT g6<br>01 ○ Oui<br>02 ○ Non
Effectuez-vous des tâches pour lesquelles vous n'avez pas été préparé(e) au cours de vos formations de base et complémentaire ?<br>I NE SUGGÉREZ PAS DE RÉPONSE	SINGLE-SELECT e8<br>01 ○ Oui, chaque jour<br>02 ○ Souvent<br>03 ○ Rarement<br>04 ○ Jamais<br>99 ○ Je préfère ne pas le dire
Quelles tâches pour lesquelles vous n'avez pas reçu de formation effectuez-vous en ce moment ?<br>I Plusieurs choix possibles NE PAS SUGGÉRER DE RÉPONSE<br>E e8==1 || e8==2	MULTI-SELECT e9<br>01 ☐ Consultation externe<br>02 ☐ Accident et urgence<br>03 ☐ Opération mineure<br>04 ☐ Vaccination<br>05 ☐ Services de santé maternelle/conseil<br>06 ☐ Services de planning familial/conseil<br>07 ☐ Pharmacie<br>97 ☐ Autre, précisez
Précisez autre<br>E e9.Contains(97)	TEXT e9_oth<br>..
En somme, vous sentez-vous assez bien préparé(e) et formé(e) pour votre poste actuel ?<br>I NE SUGGÉREZ PAS DE RÉPONSE	SINGLE-SELECT e10<br>01 ○ Oui<br>02 ○ Parfois je me sens surchargé(e)<br>03 ○ Non, pas du tout

E TRAVAIL ACTUEL

ACTIVITÉS ET RESPONSABILITÉS

Consultez-vous des patient(e)s tous les jours ?	SINGLE-SELECT e11<br>01 ○ Oui, chaque jour<br>02 ○ Souvent<br>03 ○ Rarement<br>04 ○ Jamais
Pourquoi pas?<br>E e11==4	SINGLE-SELECT e12<br>01 ○ Pas assez de patients<br>02 ○ J'effectue d'autre tâches<br>97 ○ Autre, précisez

Précisez autre<br><br>E e12==97	TEXT e12oth<br>........................
En moyenne, combien de patient(e)s consultez-vous chaque jour lorsque vous êtes dans cet établissement ?<br><br>E e11==1 || e11==2 || e11==3<br>V1 self.InRange(1,80)<br>M1 Nombre non-valide	NUMERIC: INTEGER e13<br>_ _ _ _ _ _ _ _ _ _ _ _ _ _ _ _ _ _ _ _
Que pensez-vous du nombre de patient(e)s que vous voyez tous les jours ?<br><br>I SUGGÉRER DE RÉPONSE<br>E e11==1 || e11==2 || e11==3	SINGLE-SELECT e14<br>01 ○ C'est plus que je ne peux gérer<br>02 ○ Acceptable<br>03 ○ Je pourrais en gérer un peu plus<br>04 ○ Je pourrais en gérer beaucoup plus
Répondez-vous à tous les attentes médicales des patient(e)s que vous consultez ?<br><br>E e11==1 || e11==2	SINGLE-SELECT e15<br>01 ○ Oui<br>02 ○ Non
Pourquoi pas?<br><br>I NE SUGGÉREZ PAS DE RÉPONSE<br>E (e11==1 || e11==2 || e11==3) && e15==2	SINGLE-SELECT e16<br>01 ○ Nous n'offrons qu'un service limités<br>02 ○ Manque de matériel<br>03 ○ Materiel non fonctionnel<br>04 ○ Manque de formation/compétences<br>05 ○ Manque de personnel<br>97 ○ Autre, précisez
Précisez autre<br><br>E (e11==1 || e11==2 || e11==3) && e16==97	TEXT e16oth<br>........................

E TRAVAIL ACTUEL

CHARGE DE TRAVAIL

Au cours d'une semaine normale, combien de jours travaillez-vous dans cet établissement ?<br><br>I Écrivez le nombre de jours par semaine. Écrivez "0" si vous ne travaille z pas dans cet établissement.<br>V1 self.InRange(0,7)<br>M1 Nombre non-valide	NUMERIC: INTEGER e17<br>_ _ _ _ _ _ _ _ _ _ _ _ _ _ _ _ _ _ _ _
Avez-vous l'impression que vos collègues (même cadre) travaillent environ le même nombre de jours ?	SINGLE-SELECT e18<br>01 ○ Plus<br>02 ○ Même<br>03 ○ Moins<br>04 ○ NA
Au cours d'une journée normale, combien d'heures travaillez-vous dans cet établissement ?<br><br>I Écrivez le nombre d'heures par jour.<br>V1 self.InRange(0,24)<br>M1 Nombre non-valide	NUMERIC: INTEGER e19<br>_ _ _ _ _ _ _ _ _ _ _ _ _ _ _ _ _ _ _ _
Avez-vous l'impression que vos collègues (même cadre) travaillent environ le même	SINGLE-SELECT e20<br>01 ○ Plus

nombre d'heures dans une journée ?	02 ○ Même<br>03 ○ Moins<br>99 ○ NA
Pensez-vous que votre emploi du temps vous permet d'accomplir aisément toutes vos tâches ?	SINGLE-SELECT e21<br>01 ○ Oui<br>02 ○ Parfois, il n'y a pas assez de temps<br>03 ○ Non, j'arrive à peine à effectuer toutes mes tâches

E TRAVAIL ACTUEL

GESTION ET CONTRÔLE

Existe-il une hiérarchie stricte dans votre établissement ?<br>I Donnez des exemples de direction stricte ou plus tolérante.	SINGLE-SELECT e22<br>01 ○ Oui<br>02 ○ Non
Vos supérieurs sont-ils généralement présents dans l'établissement de santé où vous travaillez actuellement ?	SINGLE-SELECT e23<br>01 ○ Oui<br>02 ○ Non<br>97 ○ Je ne sais pas
Que ferait votre supérieur si vous vous absentiez du travail pendant plusieurs jours ?<br>I Selectioner deux	MULTI-SELECT e24<br>01 ☐ Il/Elle essayerait de savoir ce qui se passe<br>02 ☐ Il/Elle ferait des remontrances<br>03 ☐ Il/Elle me donnerait des conseils pour m'améliorer<br>04 ☐ Il/Elle m'avertirait des conséquences<br>05 ☐ Je n'aurais pas mon salaire<br>06 ☐ Il/elle ferait des sanctions (avertissement, blâme, suspension, mis à pied)<br>07 ☐ Je serai remplacé de ma poste<br>08 ☐ Rien<br>97 ☐ Autre, précisez
Précisez autre<br>E e24.Contains(97)	TEXT e24oth<br>..
Pensez-vous que vos suggestions sont écoutées par vos supérieurs(e)s ?	SINGLE-SELECT e25<br>01 ○ Oui<br>03 ○ Rarement<br>02 ○ Non<br>97 ○ Je ne sais pas

E TRAVAIL ACTUEL

RÉFÉRENCES ETT QUALITÉ DES SERVICES RENDUS

Où referrez-vous les patients si besoin est ?	SINGLE-SELECT e26<br>01 ○ Hôpital national<br>02 ○ Hôpital régional<br>03 ○ Hôpital préfectoral<br>04 ○ Centre médico-communal<br>05 ○ Centre de santé amélioré<br>06 ○ Centre de santé<br>08 ○ Etablissement privé
Pensez-vous que votre structure sanitaire	SINGLE-SELECT e27

répond aux attentes medicales des patients ?	01 ○ Oui<br>02 ○ Rarement<br>03 ○ Non
Si vous pouviez améliorer certains aspects dans votre établissement, lequel serait le plus important ?<br><br>I SUGGÉRER DE RÉPONSE	SINGLE-SELECT e28<br>01 ○ Compétences du personnel<br>02 ○ Equipements et fournitures<br>03 ○ Médicaments et consommables<br>04 ○ Propreté et hygiène<br>05 ○ Effectifs du personnel<br>06 ○ Infrastructure<br>97 ○ Autre, précisez
Précisez autre<br><br>E e28==97	TEXT e28oth<br>..

F PRÉFÉRENCES

Qu'est-ce qui vous a motivé à travailler dans la santé ?<br><br>I NE SUGGÉREZ PAS DE RÉPONSE	SINGLE-SELECT f1<br>01 ○ Avoir un travail<br>02 ○ Avoir un emploi garanti/ la stabilité d'emploi<br>03 ○ Avoir un travail qui paie bien<br>04 ○ Avoir accès à d'autres sources de revenu<br>05 ○ Avoir un meilleur statut social<br>06 ○ Avoir une moindre charge de travail<br>07 ○ Aider les autres<br>08 ○ Continuer la tradition familiale<br>09 ○ Avoir de grandes responsabilités<br>97 ○ Autre, précisez
Précisez autre<br><br>E f1==97	TEXT f1oth<br>..
Souhaiteriez-vous changer de poste dans un prochain futur ? (Dans les 1-2 prochaines années)	SINGLE-SELECT f2<br>01 ○ Oui<br>02 ○ Non
Souhaiteriez-vous changer de profession à long terme ? (Dans 3-5 ans)	SINGLE-SELECT f4<br>01 ○ Oui<br>02 ○ Non
Pour quelle raison ?<br><br>E f4==1	SINGLE-SELECT f5<br>01 ○ Meilleur revenu/salaire<br>02 ○ Meilleures options de carrière<br>03 ○ Je n'aime pas mon travail<br>04 ○ Trop de stress<br>05 ○ Trop de charge<br>97 ○ Autre, précisez
Précisez autre<br><br>E f5==97	TEXT f5_oth<br>..
Souhaiteriez-vous travailler à long terme, en milieu urbain ou rural ?	SINGLE-SELECT f3<br>01 ○ Urbain

	02 ○ Rural
À long terme, dans quel secteur souhaitez-vous travailler ?<br><br>E `f4==2`	SINGLE-SELECT f6<br>01 ○ Gouvernement/secteur public<br>02 ○ Secteur privé<br>03 ○ ONG<br>04 ○ Secteur religieux
À long terme (3-5 ans), dans quel type d'établissement souhaitez-vous travailler ?<br><br>E `f4==2`	SINGLE-SELECT f7<br>01 ○ Hôpital national<br>02 ○ Hôpital régional<br>03 ○ Hôpital préfectoral<br>04 ○ Centre médico-communal<br>05 ○ Centre de santé amélioré<br>06 ○ Centre de santé<br>07 ○ Poste de santé
À long terme (3-5 ans), souhaiteriez-vous travailler dans la domaine de la sante à l'étranger ?	SINGLE-SELECT f8<br>01 ○ Oui<br>02 ○ Non

G ANTÉCÉDENTS PROFESSIONNELS

Depuis combien d'années travaillez-vous dans votre profession ?<br><br>I Écrivez le nombre total d'années. En cas de moins d'un an, écrivez 0<br>V1 `self.InRange(0,70)`<br>M1 Numbre non-valide	NUMERIC: INTEGER g1<br>_ _ _ _ _ _ _ _ _ _ _ _ _ _ _ _ _ _ _ _
Avez-vous travaillé comme professionnel de la santé en dehors de la Guinée ?	SINGLE-SELECT g2<br>01 ○ Oui<br>02 ○ Non
Pendant combien de temps ?<br><br>I Écrivez le nombre total d'années. En cas de moins d'un an, écrivez 0<br>E `g2==1`<br>V1 `self.InRange(0,70)`<br>M1 Numbre non-valide	NUMERIC: INTEGER g3<br>_ _ _ _ _ _ _ _ _ _ _ _ _ _ _ _ _ _ _ _
Où ?<br><br>I Si c'est le cas pour plusieurs pays, écrire le pays dans lequel le séjour professionnel était le plus long.<br>E `g2==1`	TEXT g4<br>..
Depuis combien d'années travaillez-vous dans votre profession en Guinée ?<br><br>I Écrivez le nombre total d'années. En cas de moins d'un an, écrivez 0<br>E `g2==1`<br>V1 `self.InRange(0,70)`<br>M1 Numbre non-valide	NUMERIC: INTEGER g5<br>_ _ _ _ _ _ _ _ _ _ _ _ _ _ _ _ _ _ _ _

STATIC TEXT

Cours de formation

Travailliez-vous en ce moment à proximité de l'endroit où vous avez recu votre formation professionnelle ?	SINGLE-SELECT g7<br>01 ○ Oui<br>02 ○ Non

Travailliez-vous en ce moment dans la région où vous avez grandi ?	SINGLE-SELECT g8<br>01 ○ Oui<br>02 ○ Non

STATIC TEXT

Enumerator pour donner des exemples de zones urbaines et rurales. S'assurer que le répondant comprend les catégories

Avez-vous travaillé dans des établissements de santé en zone urbaine ?	SINGLE-SELECT g9<br>01 ○ Oui<br>02 ○ Non
Pendant combien d'années ?<br>I Écrivez le nombre total d'années. En cas de moins d'un an, écrivez 0<br>E `g9==1`<br>V1 `self.InRange(0,70)`<br>M1 Numbre non-valide	NUMERIC: INTEGER g10<br>_ _
Avez-vous travaillé dans des établissements de santé en zone rurale ?	SINGLE-SELECT g11<br>01 ○ Oui<br>02 ○ Non
Pendant combien d'années ?<br>I Écrivez le nombre total d'années. En cas de moins d'un an, écrivez 0<br>E `g11==1`<br>V1 `self.InRange(0,70)`<br>M1 Numbre non-valide	NUMERIC: INTEGER g12<br>_ _

STATIC TEXT

L'enquêteure doit donner des exemples locaux d'hôpital national, d'hôpital régional, d'hôpital préfectoral, de centres médico-communaux, de centres de santé et de postes de santé que la personne interrogée peut connaître. Veuillez à ce que la personne comprenne les catégories.

Avez-vous travaillé dans les hôpitaux nationaux ?	SINGLE-SELECT g13<br>01 ○ Oui<br>02 ○ Non
Pendant combien d'années ?<br>I Écrivez le nombre total d'années. En cas de moins d'un an, écrivez 0<br>E `g13==1`<br>V1 `self.InRange(0,70)`<br>M1 Numbre non-valide	NUMERIC: INTEGER g14<br>_ _
Avez-vous travaillé dans les hôpitaux régionaux?	SINGLE-SELECT g15<br>01 ○ Oui<br>02 ○ Non
Pendant combien d'années ?<br>I Écrivez le nombre total d'années. En cas de moins d'un an, écrivez 0<br>E `g15==1`<br>V1 `self.InRange(0,70)`<br>M1 Numbre non-valide	NUMERIC: INTEGER g16<br>_ _
Avez-vous travaillé dans les hôpitaux préfectoraux ou dans les centres médico-communaux ?	SINGLE-SELECT g17<br>01 ○ Oui<br>02 ○ Non
Pendant combien d'années ?<br>I Écrivez le nombre total d'années. En cas de moins d'un an, écrivez 0	NUMERIC: INTEGER g18<br>_ _

E g17==1<br>V1 self.InRange(0,70)<br>M1 Numbre non-valide	
Avez-vous travaillé dans les centres de santé ?	SINGLE-SELECT g19<br>01 ○ Oui<br>02 ○ Non
Pendant combien d'années ?<br>I Écrivez le nombre total d'années. En cas de moins d'un an, écrivez 0<br>E g19==1<br>V1 self.InRange(0,70)<br>M1 Numbre non-valide	NUMERIC: INTEGER g20<br>_ _ _ _ _ _ _ _ _ _ _ _ _ _ _ _ _ _ _ _
Avez-vous travaillé dans les postes de santé ?	SINGLE-SELECT g21<br>01 ○ Oui<br>02 ○ Non
Pendant combien d'années ?<br>I Écrivez le nombre total d'années. En cas de moins d'un an, écrivez 0<br>E g21==1<br>V1 self.InRange(0,70)<br>M1 Numbre non-valide	NUMERIC: INTEGER g22<br>_ _ _ _ _ _ _ _ _ _ _ _ _ _ _ _ _ _ _ _
En tout, combien de fois avez-vous changé de structure sanitaire au cours de votre carrière en Guinée ?<br>I Écrivez le nombre de transferts. Écrire "0" si aucun transfert n'a eu lie u.	NUMERIC: INTEGER g23<br>_ _ _ _ _ _ _ _ _ _ _ _ _ _ _ _ _ _ _ _
En tout, combien de fois avez-vous changé de postes au cours de votre carrière en Guinée ?<br>I Écrivez le nombre de transferts. Écrire "0" si aucun transfert n'a eu lie u.	NUMERIC: INTEGER g24<br>_ _ _ _ _ _ _ _ _ _ _ _ _ _ _ _ _ _ _ _
Avez-vous travaillé dans des établissements de santé privés ?	SINGLE-SELECT g25<br>01 ○ Oui<br>02 ○ Non
Pendant combien d'années ?<br>I Écrivez le nombre total d'années. En cas de moins d'un an, écrivez 0<br>E g25==1<br>V1 self.InRange(0,70)<br>M1 Numbre non-valide	NUMERIC: INTEGER g26<br>_ _ _ _ _ _ _ _ _ _ _ _ _ _ _ _ _ _ _ _

H REVENU ET DÉPENSES

H REVENU ET DÉPENSES

REVENU

Quel est votre salaire brut par mois ?<br>I Écrire le montant en milliers de FG 99998 Je ne sais pas<br>V1 self.InRange(0,20000) || self==99998<br>M1 Numbre non-valide	NUMERIC: INTEGER h1<br>_ _ _ _ _ _ _ _ _ _ _ _ _ _ _ _ _ _ _ _
Quel est votre salaire net par mois ?	NUMERIC: INTEGER h2

I Écrire le montant en milliers de FG 99998 Je ne sais pas<br>V1 `self.InRange(0,20000) || self==99998`<br>M1 Nombre non-valide	_ _

STATIC TEXT

Au cours d'un mois normal, combien recevez-vous des paiements supplémentaires officiels suivants?

INSTRUCTION
Écrire le montant en milliers GNF. Enregistrez le montant de la fiche de paie
Écrirez '0' si aucun

Aide au transport<br>V1 `self.InRange(0,2000)`<br>M1 Nombre non-valide	NUMERIC: INTEGER h7<br>_ _
Aide au logement<br>V1 `self.InRange(0,2000)`<br>M1 Nombre non-valide	NUMERIC: INTEGER h6<br>_ _
Autres indemnités régulières ?<br>V1 `self.InRange(0,2000)`<br>M1 Nombre non-valide	NUMERIC: INTEGER h8<br>_ _
Primes de risque ?<br>V1 `self.InRange(0,2000)`<br>M1 Nombre non-valide	NUMERIC: INTEGER h10<br>_ _
Indemnité journalière/ per diems<br>V1 `self.InRange(0,2000)`<br>M1 Nombre non-valide	NUMERIC: INTEGER h12<br>_ _
Paiements basés sur la performance/ paiements incitatifs<br>V1 `self.InRange(0,2000)`<br>M1 Nombre non-valide	NUMERIC: INTEGER h14<br>_ _
Recevez-vous d'autres indemnités?<br>V1 `self.InRange(0,2000)`<br>M1 Nombre non-valide	NUMERIC: INTEGER h15<br>_ _
Précisez le but<br>E `h15>0`	TEXT h16<br>...
Dans les 12 derniers mois, combien de fois votre salaire a-t-il été payé en retard ?<br>I Écrire 0, si le salaire a toujours été reçu à temps<br>V1 `self.InRange(0,12)`<br>M1 Nombre non-valide	NUMERIC: INTEGER h4<br>_ _
Dans les 12 derniers mois, combien de fois le salaire n'a-t-il pas été payé ?<br>I Écrire 0 si le salaire a toujours été reçu<br>V1 `self.InRange(0,12)`<br>M1 Nombre non-valide	NUMERIC: INTEGER h5<br>_ _

H REVENU ET DÉPENSES

Question	Réponse
Dans les établissements publics, est-ce une pratique courante que les clients paient directement aux agents de santé pour les services?	SINGLE-SELECT h17<br>01 Oui<br>02 Non
Les prix sont-ils affichés de façon visible pour les patients?<br>E h17==1	SINGLE-SELECT h17a<br>01 Oui<br>03 Certains<br>02 Non
Les patients sont-ils informés d'avance qu'il y a ces prix à payer?<br>E h17==1	SINGLE-SELECT h17b<br>01 Oui<br>02 Non
Est-ce normal, dans le établissements de santé publics, de faire payer les patients pour améliorer our accélérer les services rendus?	SINGLE-SELECT h17c<br>01 Oui<br>03 Rarement<br>02 Non<br>98 Je ne veux pas dire
En une semaine normale, quel revenu un professionnel de la santé peut-il gagner des contributions de patients dans un établissement public ?<br>I Écrire le montant en milliers de FG<br>E h17==1<br>V1 self.InRange(0,5000)<br>M1 Nombre non-valide	NUMERIC: INTEGER h18<br>_ _ _ _ _ _ _ _ _ _
Comment voyez-vous les contributions faites par les patients ?<br>I NE SUGGÉREZ PAS DE RÉPONSE<br>E h17==1	SINGLE-SELECT h19<br>01 Ce n’est pas juste / Je ne me sens<br>02 C'est toléré / c'est normal ici<br>03 Les contributions sont importantes / Elles sont méritées<br>97 Autre, précisez
Précisez autre<br>E h17==1 && h19==97	TEXT h19oth<br>....................................
Dans les établissements de santé publics, et-il courant de recevoir des cadeaux en nature de la part des patients ?	SINGLE-SELECT h20<br>01 Oui<br>02 Non
Quel genre de cadeaux en nature les patients apportent, ils aux professionnels de santé ?<br>I NE SUGGEREZ PAS DE REPONSE. PLUSIEURS RÉPONSES POSSIBLES<br>E h20==1	MULTI-SELECT h21<br>01 Légumes/denrées de base/Fruits/Viande/Produits laitiers<br>02 Tissu<br>03 Cartes de crédit du portable<br>04 Produits de beauté/savon<br>97 Autre, précisez
Précisez autre<br>E h20==1 && h21.Contains(97)	TEXT h21oth<br>....................................
Quels sont vos sentiments à ce sujet ?	SINGLE-SELECT h23

I NE SUGGÉREZ PAS DE RÉPONSE<br>E `h20==1`	01 ○ Ce n'est pas juste / Je ne me sens<br>02 ○ C'est toléré / c'est normal ici<br>03 ○ Les contributions sont importantes / Elles sont méritées<br>97 ○ Autre, précisez
Précisez autre<br>E `h23==97`	TEXT h23oth<br>..
Est-il courant d'offrir des consultations privées aux patients en dehors des heures de travail ?	SINGLE-SELECT h24<br>01 ○ Oui<br>02 ○ Non
Au cours d'une semaine normale, combien pensez-vous qu'on peut gagner avec ces consultations privées ?<br>I Écrire le montant en milliers de FG<br>E `h24==1`<br>V1 `self.InRange(0,50000)`<br>M1 Nombre non-valide	NUMERIC: INTEGER h25<br>_ _ _ _ _ _ _ _ _ _ _ _ _ _ _ _ _ _ _ _

H REVENU ET DÉPENSES

REVENUS SUPPLÉMENTAIRES

Est-il courant d'avoir des activités génératrices de revenus comme l'agriculture, en plus de travailler dans le secteur de la santé ?	SINGLE-SELECT h26<br>01 ○ Oui<br>02 ○ Non
Quelle importance accordez-vous au revenu du travail dans l'établissement public, comparé à d'autres sources de revenus tels que l'agriculture, l'élevage ou d'autres activités génératrices de revenus dans le ménage du professionnel de santé?<br>E `h26==1`	SINGLE-SELECT h27<br>01 ○ C'est la source principale de revenu du ménage<br>02 ○ D'autres activités telles que l'agriculture, l'élevage ou autres sont la source principale de revenus du ménage<br>03 ○ Ces activités on la même importance que le travail dand l'établissement de santé<br>97 ○ Autre, précisez
Précisez autre<br>E `h27==97`	TEXT h27oth<br>..

H REVENU ET DÉPENSES

DÉPENSES DU MÉNAGE

Vivez-vous avec votre époux(se)/partenaire ?	SINGLE-SELECT h28<br>01 ○ Oui<br>02 ○ Non
Est-ce que votre époux(se) est salarié(e) ou travaille à son compte ?<br>E `h28==1`	SINGLE-SELECT h30<br>01 ○ À son compte<br>02 ○ Salarié(e)<br>03 ○ Mon époux(se) ne travaille pas
Combien d'argent gagne votre époux(se) par semaine ?	NUMERIC: INTEGER h31

I Écrire le montant en milliers de FG 99998 Je ne sais pas<br>E `h30==1`<br>V1 `self.InRange(0,1250) || self==99998`	________________
Combien d'argent gagne votre époux(se) par mois ?<br>I Écrire le montant en milliers de FG 99998 Je ne sais pas<br>E `h30==2`<br>V1 `self.InRange(0,5000) || self==99998`	NUMERIC: INTEGER h32<br>________________
Combien de personnes contribuent au revenu mensuel de votre menage ?<br>V1 `self.InRange(0,21)`<br>M1 Nombre non-valide	NUMERIC: INTEGER h29<br>________________
Combien de personnes dépendant financièrement de vous vivent dans votre ménage ?<br>V1 `self.InRange(0,40)`	NUMERIC: INTEGER h34<br>________________
Quelle est la plus importante dépense de votre ménage ?<br>I Ne suggérez pas de réponse	SINGLE-SELECT h35<br>01 ○ Frais scolaire/uniformes<br>02 ○ Événements du cycle de la vie (marriages, baptêmes, décès par ex.)<br>03 ○ Frais médicaux/médicaments<br>04 ○ Intrants agricoles<br>05 ○ Nourriture<br>06 ○ Loyer<br>07 ○ Transport<br>98 ○ Ne veux pas répondre<br>97 ○ Autre, précisez
Précisez autre<br>E `h35==97`	TEXT h35oth<br>................................
Comment supportez-vous principalment cette dépense en gros ?<br>I NE SUGGEREZ PAS DE REPONSE.	SINGLE-SELECT h36<br>01 ○ Crédit: famille/amis<br>02 ○ Crédit: avance sur salaires<br>03 ○ Épargnes<br>04 ○ Vente de biens<br>05 ○ Frais sont couverts par mon salaire<br>06 ○ Réduction des dépenses<br>07 ○ Prêts bancaires<br>99 ○ Ne veux pas répondre<br>97 ○ Autre, précisez
Précisez autre<br>E `h36==97`	TEXT h36oth<br>................................
Votre ménage parvient-il à couvrir ces dépenses de base chaque semaine ?<br>I NE SUGGÉREZ PAS DE RÉPONSE	SINGLE-SELECT h37<br>01 ○ Oui, toujours<br>02 ○ La plupart du temps<br>03 ○ Rarement<br>04 ○ Jamais<br>99 ○ Je ne veux pas dire

H REVENU ET DÉPENSES

ALLOCATIONS AU LOGEMENT ET AU TRANSPORT

Question	Réponse
Combien de jours de congés avez-vous par an ?<br><br>I Écrivez le nombre de jours par an.<br>V1 `self.InRange(0,90)`<br>M1 Nombre non-valide	NUMERIC: INTEGER — h38<br><br>_ _
Votre emploi vous fournit-il un logement ?	SINGLE-SELECT — h39<br>01 ○ Oui<br>02 ○ Non
Quelle est l'état de ce logement ?<br><br>I Suggérez des réponses<br>E `h39==1`	SINGLE-SELECT — h40<br>01 ○ Excellent<br>02 ○ Bon<br>03 ○ Décent<br>04 ○ Pauvre<br>05 ○ Terrible
Au cours d'une semaine normale, à quelle fréquence utilisez-vous le logement qui vous est fourni ?<br><br>I Écrivez le nombre de nuits par semaine.<br>E `h39==1`<br>V1 `self.InRange(0,7)`<br>M1 Nombre non-valide	NUMERIC: INTEGER — h41<br><br>_ _
Utilisez-vous un mode de transport pour exécuter votre travail ? (par exemple, motocyclette/voiture)	SINGLE-SELECT — h42<br>01 ○ Oui<br>02 ○ Non
Recevez-vous assez de carburant ou d'aide au carburant de la part de votre établissement afin d'utiliser une motocyclette ou voiture pour votre travail ?<br><br>E `h42==1`	SINGLE-SELECT — h43<br>01 ○ Oui, toujours<br>02 ○ La plupart du temps<br>03 ○ Rarement ou jamais

I ABSENTÉISME

Question	Réponse
En général, vos collègues dans cet établissement sont-ils souvent absents au travail ?<br><br>I Plus d'un quart	SINGLE-SELECT — i1<br>01 ○ Oui<br>02 ○ Non<br>99 ○ Je ne veux pas dire
Sur un mois, combien de jours pensez-vous que vos collègues sont absents dans cet établissement ?<br><br>I 99998 Je ne sais pas<br>V1 `self.InRange(0,30) || self==99998`<br>M1 Nombre non-valide	NUMERIC: INTEGER — i2<br><br>_ _
Quelle est la raison principale, d'après vous ?<br><br>I NE SUGGEREZ PAS DE REPONSE.	SINGLE-SELECT — i3<br>00001 ○ Domicile éloigné du lieu de travail<br>00002 ○ Maladie<br>00003 ○ Membre de la famille malade<br>00004 ○ Autre emploi<br>00005 ○ Consultation privée<br>00006 ○ Raison familiale<br>00007 ○ Obligations domestiques

	00008 ○ Fatigué(e) des jours précédents<br>00097 ○ Autre, précisez<br>99998 ○ Je ne sais pas
Précisez autre<br><br>E i3==97	TEXT i3oth<br><br>..
Pensez-vous travailler plus ou moins de jours que vos collègues ?	SINGLE-SELECT i4<br>01 ○ Plus<br>02 ○ Même<br>03 ○ Moins<br>99 ○ NA
Dans les 30 derniers jours, avez-vous été absent au travail pour cause de maladie ? Combien de jours?<br><br>I Écrire le nombre de jours, indiquez 0 pour 'non'<br>V1 `self.InRange(0,30)`<br>M1 Nombre non-valide	NUMERIC: INTEGER i5<br><br>- -
Dans les 30 derniers jours, avez-vous été absent au travail pour cause de formation, sensibilisation ou congé autorisé ?<br><br>I Écrire le nombre de jours, indiquez 0 pour 'non'<br>V1 `self.InRange(0,30)`<br>M1 Nombre non-valide	NUMERIC: INTEGER i6<br><br>- -
Dans les 30 derniers jours, avez-vous été absent au travail pour d'autres raisons personnelles ? Combien de jours?<br><br>I Écrire le nombre de jours, indiquez 0 pour 'non'<br>V1 `self.InRange(0,30)`<br>M1 Nombre non-valide	NUMERIC: INTEGER i7<br><br>- -
Quelle était la raison de votre absence la dernière fois que vous avez manqué le travail pour des raisons personnelles ?<br><br>I NE SUGGÉREZ PAS DE RÉPONSE	SINGLE-SELECT i8<br>01 ○ Domicile éloigné du lieu de travail<br>02 ○ J'étais malade<br>03 ○ Membre de la famille malade<br>04 ○ Je devais aller à un autre travail<br>05 ○ Consultation privée<br>06 ○ Je dois m'occuper des enfants<br>07 ○ J'avais des tâches ménagères à faire<br>08 ○ J'étais fatigué(e) des jours précédents<br>99 ○ Jamais été abrent du travail pour des raisons personnelles<br>97 ○ Autre, précisez
Précisez autre<br><br>E i8==97	TEXT i8oth<br><br>..

J SATISFACTION ET CONTRAINTES

J SATISFACTION ET CONTRAINTES

SATISFACTION

Veuillez évaluer votre satisfaction dans les aspects suivants de votre vie en général ?<br><br>I Suggérez des réponses	SINGLE-SELECT j1a<br>01 ○ Très satisfait(e)<br>02 ○ Satisfait(e)<br>03 ○ Mécontent(e)<br>04 ○ Très mécontent(e)
Veuillez évaluer votre satisfaction dans les aspects suivants de votre situation financière ?<br><br>I Suggérez des réponses	SINGLE-SELECT j1b<br>01 ○ Très satisfait(e)<br>02 ○ Satisfait(e)<br>03 ○ Mécontent(e)<br>04 ○ Très mécontent(e)
Veuillez évaluer votre satisfaction dans les aspects suivants de votre conditions de travail ?	SINGLE-SELECT j1d<br>01 ○ Très satisfait(e)<br>02 ○ Satisfait(e)<br>03 ○ Mécontent(e)<br>04 ○ Très mécontent(e)
Veuillez évaluer votre satisfaction dans les aspects suivants de votre Équilibre entre travail et loisirs ?	SINGLE-SELECT j1c<br>01 ○ Très satisfait(e)<br>02 ○ Satisfait(e)<br>03 ○ Mécontent(e)<br>04 ○ Très mécontent(e)
Veuillez évaluer votre satisfaction dans les aspects suivants de votre opportunités de carrière ?	SINGLE-SELECT j1e<br>01 ○ Très satisfait(e)<br>02 ○ Satisfait(e)<br>03 ○ Mécontent(e)<br>04 ○ Très mécontent(e)

J SATISFACTION ET CONTRAINTES

DÉCLARATIONS ET MOTIVATION

Dans quelle mesure êtes-vous d'accord avec les phrases suivantes ? J'ai assez d'occasions pour apprendre dans mon établissement.<br><br>I Suggérez des réponses	SINGLE-SELECT j2a<br>01 ○ Complètement d'accord<br>02 ○ D'accord<br>03 ○ En désaccord<br>04 ○ Pas du tout d'accord
Les perspectives de carrière sont équitables et les promotions basées sur la performance.	SINGLE-SELECT j2b<br>01 ○ Complètement d'accord<br>02 ○ D'accord<br>03 ○ En désaccord<br>04 ○ Pas du tout d'accord
Je suis motivé(e) pour aller au travail tous les jours.	SINGLE-SELECT j2c<br>01 ○ Complètement d'accord<br>02 ○ D'accord<br>03 ○ En désaccord<br>04 ○ Pas du tout d'accord
Mon salaire est trop faible pour couvrir les dépenses de base de ma famille.	SINGLE-SELECT j2d<br>01 ○ Complètement d'accord<br>02 ○ D'accord<br>03 ○ En désaccord<br>04 ○ Pas du tout d'accord
Le personnel soignant est forcé de demander aux patients de l'argent afin de gagner sa vie.	SINGLE-SELECT j2e<br>01 ○ Complètement d'accord<br>02 ○ D'accord<br>03 ○ En désaccord

	04 ○ Pas du tout d'accord
Le chômage m'inquiète.	SINGLE-SELECT j2f<br>01 ○ Complètement d'accord<br>02 ○ D'accord<br>03 ○ En désaccord<br>04 ○ Pas du tout d'accord
Nous avons assez de médicaments et de matériel pour traiter les patients.	SINGLE-SELECT j2h<br>01 ○ Complètement d'accord<br>02 ○ D'accord<br>03 ○ En désaccord<br>04 ○ Pas du tout d'accord
J'ai peur d'être transféré(e) dans une autre région.	SINGLE-SELECT j2g<br>01 ○ Complètement d'accord<br>02 ○ D'accord<br>03 ○ En désaccord<br>04 ○ Pas du tout d'accord
Nous avons assez de matériel en bon état de marche pour traiter les patients	SINGLE-SELECT j2i<br>01 ○ Complètement d'accord<br>02 ○ D'accord<br>03 ○ En désaccord<br>04 ○ Pas du tout d'accord
Quand je fais un bon travail, je me sens plus à l'aise avec moi-même.	SINGLE-SELECT j2j<br>01 ○ Complètement d'accord<br>02 ○ D'accord<br>03 ○ En désaccord<br>04 ○ Pas du tout d'accord
Je travaille ici parce-que le salaire correspond de manière juste aux efforts de chaque professionnel de santé.	SINGLE-SELECT j2k<br>01 ○ Complètement d'accord<br>02 ○ D'accord<br>03 ○ En désaccord<br>04 ○ Pas du tout d'accord
C'est important pour moi d'avoir l'occasion d'avancer dans ma carrière.	SINGLE-SELECT j2l<br>01 ○ Complètement d'accord<br>02 ○ D'accord<br>03 ○ En désaccord<br>04 ○ Pas du tout d'accord
Je suis content de travailler dans cet établissement de santé plutôt que dans un autre.	SINGLE-SELECT j2m<br>01 ○ Complètement d'accord<br>02 ○ D'accord<br>03 ○ En désaccord<br>04 ○ Pas du tout d'accord
Je me sens personellement responsable si nous ne pouvons pas satisfaire les besoins d'un(e) patient(e).	SINGLE-SELECT j2n<br>01 ○ Complètement d'accord<br>02 ○ D'accord<br>03 ○ En désaccord<br>04 ○ Pas du tout d'accord
Je pense souvent à démissionner.	SINGLE-SELECT j2o<br>01 ○ Complètement d'accord<br>02 ○ D'accord<br>03 ○ En désaccord<br>04 ○ Pas du tout d'accord
Mes collègues sont solidaires, c'est pourquoi	SINGLE-SELECT j2p

j'aime travailler ici.	01 Complètement d'accord<br>02 D'accord<br>03 En désaccord<br>04 Pas du tout d'accord
Je travaille ici parce que cela représente une sécurité à long-terme.	SINGLE-SELECT j2q<br>01 Complètement d'accord<br>02 D'accord<br>03 En désaccord<br>04 Pas du tout d'accord
Je travaille ici parce qu'il y a des opportunités d'avancer ma carrière.	SINGLE-SELECT j2r<br>01 Complètement d'accord<br>02 D'accord<br>03 En désaccord<br>04 Pas du tout d'accord
Je sens que par mon travail, je peux accomplir quelque chose d'important	SINGLE-SELECT j2s<br>01 Complètement d'accord<br>02 D'accord<br>03 En désaccord<br>04 Pas du tout d'accord
Je travaille ici parce que cela me plait.	SINGLE-SELECT j2t<br>01 Complètement d'accord<br>02 D'accord<br>03 En désaccord<br>04 Pas du tout d'accord
C'est difficile d'insister que le travail soit bien fait.	SINGLE-SELECT j2u<br>01 Complètement d'accord<br>02 D'accord<br>03 En désaccord<br>04 Pas du tout d'accord
Parfois, j'ai l'impression que mon travail ne vaut pas la peine.	SINGLE-SELECT j2v<br>01 Complètement d'accord<br>02 D'accord<br>03 En désaccord<br>04 Pas du tout d'accord
La reconnaissance de la communauté pour le travail que je fais est importante pour moi.	SINGLE-SELECT j2w<br>01 Complètement d'accord<br>02 D'accord<br>03 En désaccord<br>04 Pas du tout d'accord

K DESCRIPTION DE LA PERSONNE INTERROGÉE

Sexe	SINGLE-SELECT k1<br>01 Homme<br>02 Femme
Quel est votre âge ?<br>I Écrire l'âge en années passées<br>V1 `self.InRange(0,80)`<br>M1 Nombre non-valide	NUMERIC: INTEGER k2<br>_ _ _ _ _ _ _ _ _ _ _ _ _ _ _ _ _ _ _ _
Quel est votre statu matrimoniale?	SINGLE-SELECT k3<br>01 Célibataire

	02 ○ Fiancé(e)<br>03 ○ Vit en partenariat<br>04 ○ Marié(e)<br>05 ○ Veuf ou veuve<br>06 ○ Divorcé/e<br>07 ○ Séparé/e
Est-ce que votre époux/se ou partenaire travaille ou étudie dans le secteur de la santé ?<br><br>E `k3.InList(2,3,4)`	SINGLE-SELECT k4<br>01 ○ Oui<br>02 ○ Non
Où habite votre époux(se) ou partenaire ?<br><br>E `k3.InList(2,3,4)`	SINGLE-SELECT k5<br>01 ○ Boké<br>02 ○ Conakry<br>03 ○ Faranah<br>04 ○ Kankan<br>05 ○ Kindia<br>06 ○ Labé<br>07 ○ Mamou<br>08 ○ Nzérékoré<br>09 ○ À l'étranger
Où?<br><br>E `k3 != 1 && k5==9`	TEXT k5oth<br>..
Combien d'enfants avez-vous?<br><br>I Indiquez '0' si aucun.	NUMERIC: INTEGER k6<br>_ _ _ _ _ _ _ _ _ _ _ _ _ _ _ _ _ _ _ _
Dans quelle région êtes-vous né(e) ?	SINGLE-SELECT k7<br>01 ○ Boké<br>02 ○ Conakry<br>03 ○ Faranah<br>04 ○ Kankan<br>05 ○ Kindia<br>06 ○ Labé<br>07 ○ Mamou<br>08 ○ Nzérékoré<br>09 ○ À l'étranger
Où?<br><br>E `k7==9`	TEXT k7oth<br>..
Dans quelle région avez-vous grandi ?	SINGLE-SELECT k8<br>01 ○ Boké<br>02 ○ Conakry<br>03 ○ Faranah<br>04 ○ Kankan<br>05 ○ Kindia<br>06 ○ Labé<br>07 ○ Mamou<br>08 ○ Nzérékoré<br>09 ○ À l'étranger
Où?<br><br>E `k8==9`	TEXT k8oth<br>..
Quel est le niveau d’instruction de votre père ?	SINGLE-SELECT k13<br>01 ○ Non-scolarisé<br>03 ○ Éducation primaire (complété)

	04 ○ Éducation secondaire (complété)<br>05 ○ Diplôme ou formation professionnelle<br>06 ○ Diplôme d'enseignement supérieur<br>99 ○ Je ne sais pas<br>97 ○ Autre, précisez
Précisez autre<br>E k13==97	TEXT k13oth<br>..
Quel est/était l'occupation principalee secteur d'activité de votre père ?	SINGLE-SELECT k14<br>01 ○ Ménagère<br>02 ○ Couturier/tailleur<br>03 ○ Commerçant/marchand<br>04 ○ Elève/étudiant<br>05 ○ Ouvrier (maçon, mécanicien, menuisier, électricien, peintre)<br>06 ○ Agriculteur<br>07 ○ Transporteur/chauffeur<br>08 ○ Eleveur<br>09 ○ Pécheur<br>10 ○ Fonctionnaire/agronome/ingénieur/enseignant<br>11 ○ Agent de santé<br>97 ○ Autre, précisez
Précisez autre<br>E k14==97	TEXT k14oth<br>..
À qui appartenait la maison dans laquelle vous avez grandi ?	SINGLE-SELECT k15<br>01 ○ Le ménage<br>02 ○ La famille du chef de famille<br>03 ○ À l'État<br>04 ○ À une société ou entreprise privée<br>05 ○ Propriétaire privé<br>97 ○ Autre, précisez
Précisez autre<br>E k15==97	TEXT k15oth<br>..
Quelle est la classe socio-économique de vos parents, selon vous ?	SINGLE-SELECT k12<br>01 ○ Riche<br>02 ○ Classe moyenne supérieure<br>03 ○ Classe moyenne inférieure<br>04 ○ Pauvre<br>05 ○ Très pauvre
Diriez-vous que la maison dans laquelle vous avez grandi se trouve en milieu urbain ou rural ?	SINGLE-SELECT k16<br>01 ○ Urbain<br>02 ○ Rural
À combien de kilomètres environ de la maison dans laquelle vous avez grandi se trouvait la route la plus proche praticable par tous les temps ?<br>I Écrire la distance approximative en kilomètres<br>V1 self.InRange(0,300)<br>M1 Nombre non-valide	NUMERIC: INTEGER k17<br>_ _ _ _ _ _ _ _ _ _ _ _ _ _ _ _ _ _ _ _

Est-ce que la maison dans laquelle vous avez grandi comportait un des éléments suivants ?	MULTI-SELECT: YES/NO `k18`<br>02 ☐ / ☐ Réfrigérateur<br>03 ☐ / ☐ Radio<br>04 ☐ / ☐ Joueur de casettes / CD<br>05 ☐ / ☐ Télévision<br>06 ☐ / ☐ Eau courante<br>07 ☐ / ☐ Électricité<br>08 ☐ / ☐ Téléphone<br>09 ☐ / ☐ Voiture<br>10 ☐ / ☐ Moto<br>11 ☐ / ☐ Vélo<br>12 ☐ / ☐ Un terrain<br>13 ☐ / ☐ Une maison
Possédez-vous (votre ménage) un des objets suivants?	MULTI-SELECT: YES/NO `k9`<br>02 ☐ / ☐ Réfrigérateur<br>03 ☐ / ☐ Radio<br>04 ☐ / ☐ Joueur de casettes / CD<br>05 ☐ / ☐ Télévision<br>06 ☐ / ☐ Eau courante<br>07 ☐ / ☐ Électricité<br>08 ☐ / ☐ Téléphone<br>09 ☐ / ☐ Voiture<br>10 ☐ / ☐ Moto<br>11 ☐ / ☐ Vélo<br>12 ☐ / ☐ Un terrain<br>13 ☐ / ☐ Une maison
Quelle est la classe socio-économique de votre ménage, selon vous ?	SINGLE-SELECT `k12a`<br>01 ○ Riche<br>02 ○ Classe moyenne supérieure<br>03 ○ Classe moyenne inférieure<br>04 ○ Pauvre<br>05 ○ Très pauvre
Êtes-vous déjà allé(e) dans un autre pays en Afrique ?	SINGLE-SELECT `k10`<br>01 ○ Oui<br>02 ○ Non
Êtes-vous déjà allé(e) dans un autre pays hors d’Afrique ?	SINGLE-SELECT `k11`<br>01 ○ Oui<br>02 ○ Non

L FIN DE L’ENTRETIEN

Heure de fin de l’entretien	DATE: CURRENT TIME `l1`<br>..
Résultat de l’entretien	SINGLE-SELECT `l2`<br>01 ○ Complet<br>02 ○ Incomplet<br>97 ○ Autre, précisez
Résultat de l’entretien, autre précisez ?<br>E `l2==97`	TEXT `l2_oth`<br>..

Les commentaires de recenseur

TEXT 13

LEGEND

Legend and structure of information in this file

Name of section

Enabling condition for this section

Question title

Type of question, scope

Variable name

Answer options

SECTION 5: OTHER INCOME SOURCES

E s4_other_sources_which.Contains(98)

Duis aute irure dolor in reprehenderit in voluptate velit esse cillum dolore eu fugiat nulla pariatur?

MULTI-SELECT
SCOPE: PREFILLED

s4_rel_leaders_other

I **This refers to family relations**
E s3_time_other > 0
V1 s4_rel_leaders_which.Contains(98)
M1 **Can not be itself**
V2 (s3_time_other_breeding_advice <= (50 - s3_time_art_insem_advice)) ||s3_time_other_breeding_advice == 0
M2 **This person is not in the list**
F optioncode != s5_ignored_option_code

01 ☐ Community animal health workers
02 ☐ Private
03 ☐ Government
04 ☐ Livestock keepers association
05 ☐ NGO

And 5 other [13]

Additional information:
"I" — Question instruction
"E" — Enabling condition
"V1" — Validation condition №1
"M1" — Message for validation №1
"F" — Filter in Categorical questions

Link to full set in appendix

Breadcrumbs

Type or roster

Roster Title

CHAPTER 3 IDENTIFICATION /
Roster: **LEADER RELATION DETAILS**
generated by fixed list:

01 Ward Livestock Officer
02 Village Livestock Officer
99 Other (specify)

List items
