# Supplementary material for: How to attract and retain health workers in rural areas of a fragile state: Findings from a labour market survey in Guinea
Source: PLoS One. 2021 Dec 16;16(12):e0245569. doi: 10.1371/journal.pone.0245569 (PMC8675729; doi:10.1371/journal.pone.0245569)
Supplement: S3 File — (DOCX) [file pone.0245569.s003.docx]

Users’ perspectives

Our patient sample was more balanced than for health workers, with 47% of patients living in rural areas. The high socio-economic need of the population was illustrated by the fact that 67% of all patients could read or write and had not completed primary education (80% of patients in rural areas have not completed education). 74% in rural areas described themselves as poor or very poor; in urban areas this was still 53%.

A quarter of the patients lived within 1 km of the health facility and the median distance from the facility was 3 km. Looking at socio-economic status, distance tended to have a correlation with the patients’ wealth, as those in the lowest socio-economic quintile travelled an average of 25 km, the middle quintile 12 km, and the highest quintile 8 km. The larger travelling distances were often related to the patients from rural areas travelling to urban tertiary hospitals. On average, patients from rural areas travelled 21 km, while urban patients travel 8 km. Most rural patients walked to their health facility (58%), while urban patients were most likely to use public transport (61%). The mean duration of travel was 41 minutes, ranging from one minute to three hours.

The waiting time was less than an hour for 77% of the patients and within one to three hours for 21% patients. Very few patients (7 out of 485) had to wait for longer than three hours before the consultation. Those with the lowest socio-economic status tended to wait less and this could be due to wealthier patients living in urban areas where facilities are busier. Patients from rural areas were seen within the hour 81% of the time, compared to 73% from urban areas.

Consultation times varied across rural and urban areas – 15 minutes on average in the former and 21 in the later. Patients reported high scores on most domains of quality, with little difference between rural and urban areas. Patient satisfaction was high for all domains – overall satisfaction was 93% in rural areas and 89% in urban – and most would recommend the facility to friends and family. Patients were most satisfied with the language spoken, gender, and attitude of the health professional, and with laboratory investigations and physical examinations. They were least satisfied with the cost of treatment, the explanation of the illness, and taking the history of the illness.

71% of patients made some forms of payments at the health facilities, with a median payment of US$8 for those who could recollect amounts. Comparing patients coming from rural versus urban areas, 76% of patients living in urban areas had to pay fees, compared to 66% of those living in rural areas. However, the median total fee was lower for urban dwellers at US$7, compared to US$8 for patients from rural areas. There did not appear to be any significant trend across socio-economic status.

Only 27% received a receipt for their payment (31% in urban areas; 21% in rural). 2% reported paying a member of staff for better service and 2% reported providing gifts to staff. Most paid using household income (72% in rural areas, 28% in urban) and close to zero households were protected by cost sharing mechanisms such as insurance (0.7% in rural areas, 0.5% in urban). The second strategy was to rely on savings (17% of rural patients, 26% urban). However, some had to adopt riskier strategies, such as sale of assets (5% of rural, 1% urban) and loans from family or friends (10% of rural, 9% urban). According to the patients, in the future they would apply the same strategies, though with a higher emphasis on drawing on savings, taking loans, and selling assets.

Health facilities

The findings on facility functioning highlighted the unequal distribution of resources, with facilities in rural areas more likely to be poor or average in their functioning (none were found in the better functioning third of the sample). Equally, regional patterns were clear, with less well functioning facilities predominant in upper Guinea. These findings reflect that the higher-level facilities were more likely to be better performing.
