# Supplementary material for: How to attract and retain health workers in rural areas of a fragile state: Findings from a labour market survey in Guinea
Source: PLoS One. 2021 Dec 16;16(12):e0245569. doi: 10.1371/journal.pone.0245569 (PMC8675729; doi:10.1371/journal.pone.0245569)
Supplement: Attachment — Submitted filename: Reponses to review 22 07 21.docx [file pone.0245569.s005.docx]

1. Please ensure the novelty of this work is visible more substantially in your revised manuscript, mainly by acknowledging the contemporary work in your research area.

Response: We have updated our references with recent articles – see response to reviewer 2 below.

Response:

 2. Please ensure that your manuscript meets PLOS ONE's style requirements, including those for file naming. The PLOS ONE style templates can be found at

[https://journals.plos.org/plosone/s/file?id=wjVg/PLOSOne_formatting_sample_main_body.pdf](about:blank) and

[https://journals.plos.org/plosone/s/file?id=ba62/PLOSOne_formatting_sample_title_authors_affiliations.pdf](about:blank)

Response:

Thanks, we have checked and have ensured compliance with these guidelines.

3. Please provide additional details regarding participant consent. In the ethics statement in the Methods and online submission information, please ensure that you have specified what type you obtained (for instance, written or verbal, and if verbal, how it was documented and witnessed). If your study included minors, state whether you obtained consent from parents or guardians. If the need for consent was waived by the ethics committee, please include this information.

Once you have amended this/these statement(s) in the Methods section of the manuscript, please add the same text to the “Ethics Statement” field of the submission form (via “Edit Submission”).

Response: we have added these details both to the section and to the online submission form. The consent was in writing. No minors were included.

4. We note that you have reported significance probabilities of 0 in places. Since p=0 is not strictly possible, please correct this to a more appropriate limit, eg 'p<0.0001'.

Response: Thank you for this picking up this error. We have revised the p-values to <0.001 in Table 1 (now Table 3), Table 3 (now Table 5) and Table 4 (now Table 6). While revising the tables, we have also noticed that we have used asterisk symbols (*) to indicate the level of statistical significance in Table 1 (now Table 3) and Table 3 (now Table 5), but did not do so in Table 4 (now Table 6). For consistency, we have now added that in table.

5. Please include additional information regarding the survey or questionnaire used in the study and ensure that you have provided sufficient details that others could replicate the analyses. For instance, if you developed a questionnaire as part of this study and it is not under a copyright more restrictive than CC-BY, please include a copy, in both the original language and English, as Supporting Information.

Response: we have uploaded as supplementary information our questionnaires in English and French.

6. Please provide some clarification whether the ethics approval was obtained for the preliminary qualitative work conducted.

Response: We have now clarified this in the text – the approval by OPM preceded the development of tools, which included preliminary qualitative fieldwork to help with calibration, but the approval in Guinea was sought once the tools were developed and prior to more structured fieldwork.

7. Please ensure that you refer to Figures 7 & 8 in your text as, if accepted, production will need this reference to link the reader to the figures.

Response: figures 7 and 8 are referred to on page 29, in the text and also with captions.

8. Thank you for stating the following in the Competing Interests section:

[The authors have declared that no competing interests exist.].

We note that one or more of the authors are employed by a commercial company: Oxford Policy Management

 i. Please provide an amended Funding Statement declaring this commercial affiliation, as well as a statement regarding the Role of Funders in your study. If the funding organization did not play a role in the study design, data collection and analysis, decision to publish, or preparation of the manuscript and only provided financial support in the form of authors' salaries and/or research materials, please review your statements relating to the author contributions, and ensure you have specifically and accurately indicated the role(s) that these authors had in your study. You can update author roles in the Author Contributions section of the online submission form.

Please also include the following statement within your amended Funding Statement.

“The funder provided support in the form of salaries for authors [insert relevant initials], but did not have any additional role in the study design, data collection and analysis, decision to publish, or preparation of the manuscript. The specific roles of these authors are articulated in the ‘author contributions’ section.”

If your commercial affiliation did play a role in your study, please state and explain this role within your updated Funding Statement.

 ii. Please also provide an updated Competing Interests Statement declaring this commercial affiliation along with any other relevant declarations relating to employment, consultancy, patents, products in development, or marketed products, etc.

 Within your Competing Interests Statement, please confirm that this commercial affiliation does not alter your adherence to all PLOS ONE policies on sharing data and materials by including the following statement: "This does not alter our adherence to  PLOS ONE policies on sharing data and materials.” (as detailed online in our guide for authors [http://journals.plos.org/plosone/s/competing-interests](about:blank)) . If this adherence statement is not accurate and  there are restrictions on sharing of data and/or materials, please state these. Please note that we cannot proceed with consideration of your article until this information has been declared.

Please include both an updated Funding Statement and Competing Interests Statement in your cover letter. We will change the online submission form on your behalf.

Response: we have added the following to the competing interests section:

‘’This study was funded by a grant from the World Bank. The grant provided support in the form of salaries for the authors, including authors directly employed by the World Bank, and the larger study team, and also funded all field work related expenses. Aside from funding individuals involved in the study, the grant was also used to hire Oxford Policy Management, an international development consulting firm, to support overall study design, data collection and analysis. The specific roles of all authors are articulated in the ‘author contributions’ section.”

We have added that: ‘Affiliation with the World Bank, Oxford Policy Management, or any other entity involved in the study, does not alter our adherence to PLOS ONE policies in sharing data and materials’.

We have also added a disclaimer from the World Bank side:

‘The findings, inter­pretations, and conclusions expressed in this work do not necessarily reflect the views of The World Bank, its Board of Executive Directors, or the governments they represent. The World Bank does not guarantee the accuracy, completeness, or currency of the data included in this work and does not assume responsibility for any errors, omissions, or discrepancies in the information, or liability with respect to the use of or failure to use the information, methods, processes, or conclusions set forth. The boundaries, colors, denominations, and other information shown on any map in this work do not imply any judgment on the part of The World Bank concerning the legal status of any territory or the endorsement or accep­tance of such boundaries’.

Reviewer #1: Summary of study
This study uses three different nationally representative cross-sectional surveys as well as a discrete choice experiment to examine health workers’ motivations and how they could be incentivized to work in remote rural areas of Guinea. The authors examine differences by cadre of health worker as well as by location They suggest several ways to improve health worker retention in rural areas including offering specializations, recruitment from rural areas and improving working conditions.

**Major Comments**
1. While I appreciate the authors’ efforts to be thorough and to use different sources of data to examine their research question, I found the paper a little overwhelming. With results from three different surveys, a DCE, a focus on multiple outcomes disaggregated by different cadres and location, it was very difficult to determine what the main findings were of this paper. I suggest the authors simplify and split this up into multiple papers. For example, perhaps they could just focus either on the health worker survey or on the DCE in this paper. At a minimum, I think they should exclude the patient survey and health facility survey as I don’t believe they add substantially to the question of health worker motivation.

Response: thanks for this comment. While we appreciate that the article is rather dense, we do think there is value from the cross-analyses. However, we have cut the sections on the patient survey and health facility survey. We kept a summary of these as a supplementary file, as this information is relevant to the discussion, where issues of informal payment, for example, arise.

2. I think the Methods Section could use a little more detail. For example, how were the questions that are presented in Figure 2-5 asked? Were the respondents presented with responses which they had choose from? What kind of regression models were run for the analyses in Table 1 & 3 and for the DCE? The results section mentions a composite satisfaction index- how was this created? Similarly, what do the measures of intrinsic/extrinsic motivation consist of? (The authors refer to another paper, but I think a summary here would be useful).

Response: Thank you for this comment. We have included some additional texts on the type of tools and on types of the questions (e.g. prompted or unprompted).

Structured questionnaires were used in all three surveys. Unprompted response was obtained where it was required (e.g. data for Figure 2). However, the response categories were read aloud where there were no additional biases of doing so (e.g. data for Figures 3-5*).*

The type of regression used is mentioned in the analysis part of the methods section. We added that the DCE regression model was a conditional logit.

The data - [a) their confidence and reported efficacy, b) workload, c) work environment, c) financial situation, and e) four generic satisfaction scales concerning their career] - used to generate composite index is also mentioned in that section.

We have added a brief description of the approach to assigning intrinsic/extrinsic labels.

We agree that it would be great to include more details of the methodology, but at the same time we are conscious about the length of the article and have therefore tried to find a balance. We hope that this now meets expectations.


3. It would be useful to have a Table 1 that shows some descriptive statistics for the health workers surveyed; some of these statistics are cited in the text but I could not find this information in the tables.

Response: Thanks for this comment. We agree that without such a table, it might be difficult for the readers to fully understand the study population with four groups. We have now added a table with some descriptive information on the health workers (Table 2).

4. Table 4: It would be helpful if the authors could say a little more about how to interpret the coefficients in this table.

Response: This table is now table 6. We have added a little about the interpretation to facilitate the reader’s own interpretation.

5. The figures could use more labeling so that one can see what they are showing without referring to the text. For example, in Figure 2-5, it’s not clear what the question was. Figures 6 and 7 have no axis titles and, again, it’s not clear what they are showing.

Response: the figure headings for 2-5 were summaries of the question asked; we have replaced these with the working of the question. We have also improved the readability of some of the figures (e.g. the colours).

Fig 6 & 7 were updated with titles and baseline scenario colour/format change to make it stand-out. We also updated the text, adding a title in the DCE section to delimit the part where natural allocations and scenarii are developed. The baseline and scenario text has been improved, with more links to the figures.

6. If the authors choose to keep both the health worker survey and the DCE in the same paper I think it would be useful to have more discussion of to what extent the results of the health worker survey and the DCE correspond with each other.

Response: The DCE and non-DCE were designed as complimentary, rather than linked, with the non-DCE exploratory and the DCE focused on identifying policy options. As we highlight in the discussion, there were no contradictions between them. We have added a short note to this effect in the discussion.

Minor Comments
7. Table 1 and 3 are a little overwhelming: it may be helpful to have some kind of headings/categories to help orient the reader.

Response: Thank you for this comment. We agree that Table 1 and 3 (now Table 3 and 5) are full of numbers. However, it is difficult to further simplify these tables. We expect that these tables to look a bit simpler after typesetting and formatted in single line spacing.

8. Box 1: Suggest making this into a table so can see all the attributes and levels together.

Response: we have done this.

9. The description of the sampling procedure is quite complicated- perhaps a figure will help the reader?

Response: We agree that the sampling procedure was quite complicated for this study. This is because we needed to consider various different factors including regional spread, urban-rural distribution, types of facilities, randomization, ensuring equal probability, yet keeping sufficient statistical power that enables nationally representative estimates and identifying statistically significant differences between the variables of interest. We have tried to create a diagram that can illustrate this complex sampling in a simpler form, but it still requires lot of texts inside the diagram and make very little improvement in clarifying the complexity. We have therefore tried to simplify and restructure the texts in the sampling section to make it clearer. We hope this is a bit clearer.

Reviewer #2: Dear Authors,

Thank you for this paper, conducting a Labor market survey in Guinea. Actually i had already seen an earlier version and presentation of it, shared by Prof. Witter.

 My main comments are the following; This is a proper conducted health labour market analysis of the health sector , its incentives , constraints and regulation in Guinea. As a method, amongst others, A DCE has been used. Whilest this has been conducted properly, i miss consinderable 'contextual' information and 'the policy momentum' post-ebola that enabled, but also distored , workforce developments and activities in Guinea. context and health system are 'fragile' in a sense that domestic political, epidemic (Ebola, covid-19, but also new measles and polio outbreaks) as well as international actor involvement have 'shaped' the labour market in several ways. This specific Guinean governance context and momentum (post-ebola workforce investments) should be explained more explicit in introduction and discussion as it impacts specificity, relevance and policy uptake of this analysis.

Response:

Thanks, we have added more reference to the political economy factors in the discussion and the role of different actors.

As 'fragility' is in the title of the article, it must be more explicit explained what this fragility implies for the health and labour sector in Guinea

Response:

In the introduction there is a brief reference to what fragility implies for the health sector, and this issue is briefly returned to in the discussion and conclusions, but we have tried to boost, thanks,

We wrote partly about it here but several more articles should be available

Kolie, D., Delamou, A., van de Pas, R., Dioubate, N., Bouedouno, P., Beavogui, A. H., ... & Van Damme, W. (2019). ‘Never let a crisis go to waste’: post-Ebola agenda-setting for health system strengthening in Guinea. BMJ global health, 4(6), e001925.

Kolie D, van de Pas R, Delamou A, Dioubaté N, Beavogui FT, Kaba A, Beavogui AH, MD, Van De Put W, Van Damme W. Retention of Healthcare Workers one year after recruitment and deployment in rural settings: an experience Post-Ebola in five Health Districts in Guinea. Hum Resour Health. 2021
[https://doi.org/10.21203/rs.3.rs-122033/v1](about:blank)

Response:

Thanks, we have added in more recent references now.
