# Supplementary material for: How to attract and retain health workers in rural areas of a fragile state: Findings from a labour market survey in Guinea
Source: PLoS One. 2021 Dec 16;16(12):e0245569. doi: 10.1371/journal.pone.0245569 (PMC8675729; doi:10.1371/journal.pone.0245569)
Supplement: Attachment — Submitted filename: Response to second review 09 09 21.docx [file pone.0245569.s006.docx]

Many thanks to the statistical reviewer for these additional comments. Our responses are in blue below.

1. Table 1: For the time-bound contract attribute, could the authors clarify what the different levels were? Were the levels increments of a year up to the maximum of 5/7 years? Or something else?

As we say in Table 1, "*This was the maximum duration the health workers would stay at the facility before being promoted /transferred to a higher-tier facility. For medical doctors, we selected five years, and for nurses, midwives and ATS we selected seven years.*" In the context of Guinea, it would mean that health worker can yearly ask for a new location (as is the case now), however the attribute implies that a positive outcome must be obtained by the 5th/7th years. We have tried to make this clearer in the table.

2. I don’t find Table 2 particularly useful, given that it just shows the geographical distribution of the health workers. Instead (or in addition), I think it would be useful to show the descriptive statistics that are mentioned throughout the paper but aren’t shown anywhere. For example, I think the table could show summary statistics cited in the text on work/career history, workload (hours per week, patients seen), satisfaction scores, remuneration etc by health worker type. I think this would help the reader to be able to quickly see differences between the different health worker types.

We agree that, while the geographical distribution can be useful to demonstrate the national representativeness of the sample, it does not add much on understanding the study findings. We have therefore deleted that and have added some other characteristics in the table as suggested by the reviewer.

3. Are the regressions in Table 3 and Table linear models? If so, it would be good to mention this in the methods section.

Yes, we used multiple linear regression model to explain the variability in satisfaction composite score due to covariates such as sex, age etc. We have included this information in the methods section.

4. Table 3: For the satisfaction composite score- is it actually a dissatisfaction score? (it seems like a negative coefficient actually means higher satisfaction, correct?). If so, I think it would be helpful to label it as such.

We have created this composite score of satisfaction using 22 indicators, ranging from health workers’ feeling of preparedness, workload, health facility management, and working conditions, and their finances. The score ranges from 1 to 20, with higher values representing greater dissatisfaction. The negative numbers corresponding to a variable are the coefficient of the corresponding variables in the linear regression. For example, we interpreted, for variable, “is female” the negative estimate (est.) of -1.38 indicates that the dissatisfaction composite score in female is 1.3 unit less than that of males.

We have included an explanation of the construction of this composite score in the methods section. We have kept the general terminology as ‘satisfaction’ as this was the way the questions were asked, however for table 3 specifically, we take your point that the reading of the coefficients could be confusing so we have changed the column heading to clarify that the composite score is measuring dissatisfaction in effect.

5. If Table 5 presents the results of a linear model, I think the coefficients should be interpreted in terms of percentage points and not percentages.

In Table 5, we modelled variables “Currently working in rural area” and “Has long-term preference to work in rural area”, which were measured in binary scale. We have estimated the comparative likelihood of the variables for a given covariate level. For example, the covariate “has experience in rural area” was a binary variable (i.e. 0 and 1). So, the coefficient (est.) 0.146 indicates that the person who worked in rural area are 14.6% more likely to currently work in rural area.

6. In Lines 321-322, the authors say that those with higher intrinsic motivation are more likely to want to work in rural areas in the long-term, however it should be noted that this isn’t statistically significant.

We have checked but this finding is weakly significant level so we have not edited the text.
